# The gut mycobiome is shaped by interactions with the bacterial community in twins

**DOI:** 10.1016/j.isci.2026.115786

**Published:** 2026-04-16

**Authors:** Konrad Lehr, Ramiro Vilchez-Vargas, Jurgita Skieceviciene, Noam Mathias Hipler, Greta Gedgaudienė, Indre Gustaityte, Laimutis Kucinskas, Mindaugas Urba, Cosima Thon, Denny Schanze, Martin Zenker, Juozas Kupcinskas, Alexander Link

**Affiliations:** 1 Department of Gastroenterology, Hepatology and Infectious Diseases, Otto-von-Guericke University, 39120 Magdeburg, Germany; 2 Molecular Gastroenterology and Microbiome Research Unit, Institute for University Teaching and Research Medical Campus Upper Franconia, Friedrich-Alexander University Erlangen-Nürnberg, 95445 Bayreuth, Germany; 3 Institute for Digestive Research, Lithuanian University of Health Sciences, 44307 Kaunas, Lithuania; 4 Department of Gastroenterology, Lithuanian University of Health Sciences, 44307 Kaunas, Lithuania; 5 Institute of Biology Systems and Genetic Research, Lithuanian University of Health Sciences, 44307 Kaunas, Lithuania; 6 Institute of Human Genetics, Otto-von-Guericke University, 39120 Magdeburg, Germany; 7 Department of Gastroenterology, Friedrich-Alexander University Erlangen-Nürnberg, Medical Campus Upper Franconia, Klinikum Bayreuth, 95445 Bayreuth, Germany

**Keywords:** microbiome

## Abstract

The human gut microbiome comprises bacteria, viruses, and fungi, yet the fungal component (mycobiome) remains poorly characterized. Here, we investigated gut fungal composition and fungal–bacterial interactions in healthy monozygotic and dizygotic twins. Fungal communities showed substantially higher inter-individual variability than bacterial communities. Zygosity, age, and shared environment had no major influence on fungal abundance, similarity, or dominant genera. *Candida* was the most abundant genus (mean 5.2% in 161 individuals), followed by *Geotrichum* (3.7% in 132), whereas *Saccharomyces* was detected less frequently (0.8% in 92). Most bacterial genera were negatively correlated with *Candida* and *Geotrichum*, with stronger negative associations observed at higher bacterial abundances (up to rho = −0.6 for *Alistipes*). Network analysis revealed complex negative correlations among *Bacteroides*, *Prevotella*, and *Candida*. Overall, our findings reveal a highly variable gut mycobiome independent of host zygosity, pointing to a competitive bacterial-fungal interplay as a key regulator of fungal homeostasis in humans.

## Highlights

• Gut mycobiome exhibits greater heterogeneity than the bacterial community
• *Candida* and *Geotrichum* emerge as the most prevalent fungal genera
• Fungal profiles remain independent of host zygosity, aging, and shared environment
• Robust negative correlations between dominant bacterial and fungal genera

## Graphical abstract

**Figure:**
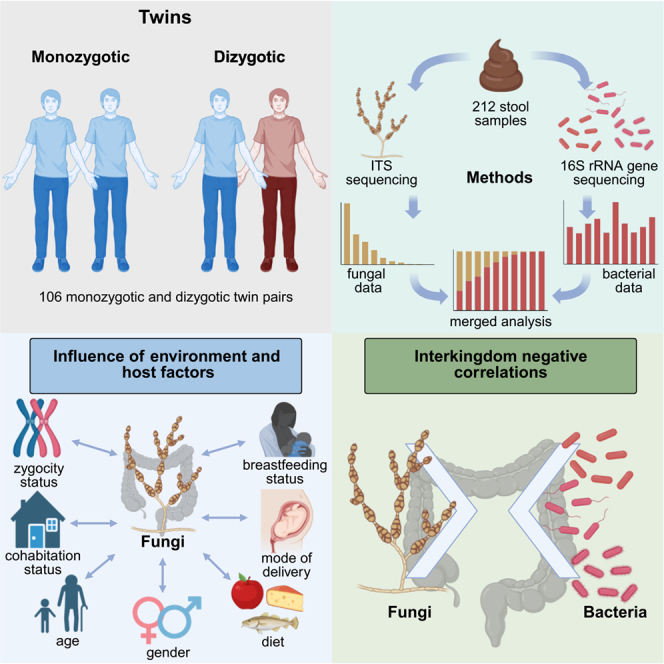

## Introduction

Advances in microbiome research have led to unprecedented insights into fundamental functional interactions within microbial ecosystems, as well as translational and clinical applications. The gut microbiota, probably the densest microbial ecosystem in the body, either functionally determines or reflects an individual’s health or disease state.^1^ Unraveling its complexity has revealed species with carcinogenic properties, such as *Helicobacter pylori* and human papillomavirus. At the same time, it has revolutionized therapeutic approaches such as fecal microbiota transplantation (FMT) for *Clostridioides difficile* infection^2^ and tumor resensitization during immunotherapy.^3^

The human microbiota consists of a wide variety of microorganisms, including viruses, archaea, fungi, and most notably, bacteria. Although significant advancements have been made in understanding the bacterial component, emerging research underscores the equally critical role of fungi.^4^ The fungal kingdom comprises up to 6 million species, yet only around 200 have been identified in humans as either commensals or pathogens.^5^ Fungi are estimated to represent less than 1% of the gut microbiome, whereas bacteria and viruses dominate, making up more than 99% of its total composition.^6^ Despite their relatively low abundance, fungi are considerably larger in size than bacteria and viruses, suggesting that they may contribute substantially to the total biomass of the intestinal microbiome.^6^ Therefore, even in smaller numbers, fungi can exert a meaningful influence on human health.

Different fungi have been linked to chronic liver diseases (*Candida albicans*),^7^ inflammatory bowel diseases (*Candida albicans*),^8^ and various cancers (*Candida, Blastomyces, Malassezia*).^9^^,^^10^ *Candida albicans*, a well-studied opportunistic pathogen, is a yeast that colonizes the human gut. It can cause gastrointestinal candidiasis and is associated with gastrointestinal inflammation, particularly due to its ability to form biofilms, which can lead to life-threatening diseases.^11^^,^^12^^,^^13^ Therefore, expanding our knowledge of this relatively understudied microbial kingdom is of growing importance.

While significant progress has been made in elucidating the dynamics of bacterial communities, understanding of the fungal community and its interaction with bacteria remains limited. Emerging data suggest that the gut mycobiome is a vital component of the microbiome, dynamically interacting with the bacterial community and correlating with health and disease states.^14^ The interplay between fungi and bacteria has been a long-standing topic of interest in controlling microbial dynamics during disease states. For example, *Lactobacillus* has been shown to alleviate gastrointestinal candidiasis in a mouse model.^15^ Similarly, *Streptococcus mutans* can inhibit *Candida* hyphal formation, preventing its attachment to epithelial cells and reducing its pathogenicity.^16^ Several other studies have demonstrated antagonistic interactions between bacteria and fungi in the human gastrointestinal tract.^17^^,^^18^^,^^19^^,^^20^^,^^21^^,^^22^

Twin studies have emerged as a unique human model to explore the interplay between genetic and environmental factors in the microbiome.^23^^,^^24^^,^^25^ Initial reports suggested that microbial similarity correlated with genetic identity, highlighting a potential heritable component in host-microbe interactions.^25^^,^^26^ However, subsequent studies have emphasized environmental factors such as cohabitation and aging as critical determinants of gut microbiome dynamics in twins.^27^^,^^28^ More recently, initial data have shed light on the virome in monozygotic (MZ) twins, providing another perspective on microbial community dynamics.^29^

In this study, we conducted a comprehensive analysis of the gut mycobiome in a well-characterized cohort of MZ and dizygotic (DZ) twins. We aimed to determine the role of genetic, phenotypic, and environmental factors for mycobiome composition in twins. We further explored the correlation between bacterial and fungal communities, assessing their interdependence and implications for health and disease.

## Results

### Fungal sequences and their interindividual similarities in a twin cohort

**Figure 1:**
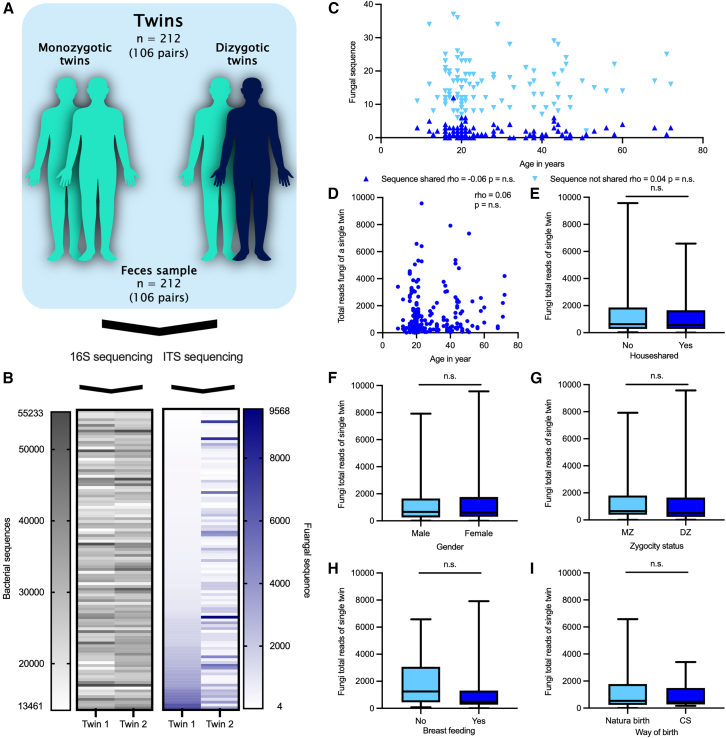
Fungal reads in a twin cohort (A) Graphical abstract of the study. (B) Heatmaps show the total number of reads (bacteria left and fungi right). Samples were sorted by increasing fungi sequence-number of twin 1. (C and D) Spearman’s correlation (rho) between (C) the number of fungal sequences shared (dark blue) and non-shared (light blue) in a twin pair, and (D) the total number of fungal sequences with age of the twins. (E–I) Association between house-shared status (E), gender (F), zygocity status (G), breastfeeding (H), and mode of birth (I) with the sum of fungal sequences in a single twin. Data are represented as mean ± standard deviation, and the Mann-Whitney test was used for testing statistical significance (E–I). None of the statistical tests reached the significance level. Please refer to Figure S1, for details of the *a priori* defined groups. MZ: monozygotic, DZ: dizygotic, CS: cesarean section.

To investigate the abundance of fungal and microbiome communities, we analyzed a cohort of 212 paired MZ and DZ twins (Figures 1A and S1). The distribution of total bacterial phylotypes was relatively high, with an abundance of at least 13,461 reads in all samples. In comparison, fungal reads showed a very heterogeneous pattern with absolute reads ranging from a minimum of 4 reads to a maximum of 9,564 reads (Figure 1B). The distribution of the low and high fungal reads was independent of age, and the shared and non-shared fungal sequences were also similarly distributed across the different ages (Figures 1C and 1D). Furthermore, systematic evaluation revealed that fungal reads did not correlate with cohabitation, gender, zygosity, breastfeeding, and mode of delivery, fat, carbohydrate, protein, or kCal intake, BMI, and birth weight (Figures 1E–1I and S2). Importantly, these findings were robust to differences in sequencing depth (≥100 reads and ≥1,000 reads), as repeating the analyses after excluding samples with low fungal read counts yielded comparable results, indicating that the observed lack of associations was not driven by low-abundance fungal profiles (Figure S3).

**Figure 2:**
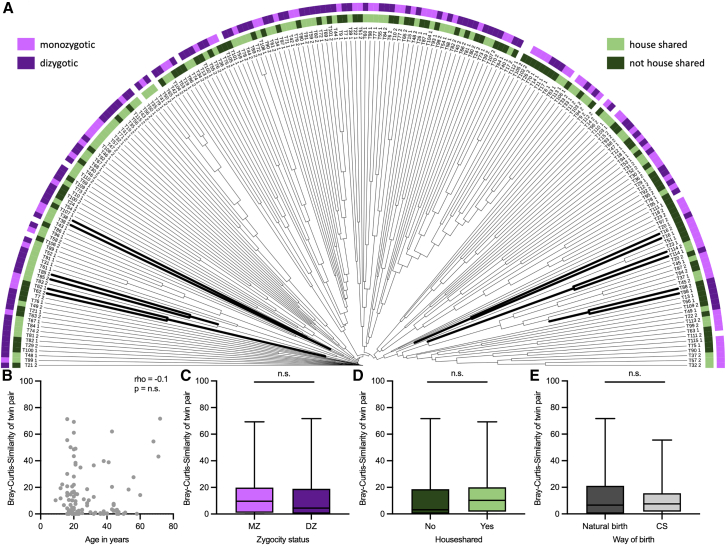
Twin similarities based on the fungal sequences (A) Hierarchical clustering of 212 fecal twin samples based on Bray-Curtis resemblance of fungal reads. Cohabitation and zygosity status are indicated. (B) Spearman correlation analysis between the Bray-Curtis resemblance of fungal reads and the age of the twins. (C–E) Comparison of the Bray-Curtis similarities of the fungal reads in twin pairs between zygosity status (C), household status (D), and way of birth (E). Data are represented as mean ± standard deviation, and the Mann-Whitney test was used for testing statistical significance (C–E). None of the statistical tests reached the significance level. Please refer to Figure S1, for details of the *a priori* defined groups. MZ: monozygotic, DZ: dizygotic, CS: cesarian section.

The overall heterogeneous fungal profile resulted in a very low average Bray-Curtis similarity of 14% for the whole cohort when compared to previously reported microbiome data.^28^ In a hierarchical cluster analysis, only 6 out of 106 twin pairs clustered directly with each other, indicating a higher similarity to each other than to the other twins (Figure 2A). This clustering was independent of zygosity and cohabitation status (Figure 2A). Bray-Curtis similarity between twin pairs was not associated with age of the twins and was similar between MZ and DZ twins, cohabitation status, different birth types, gender, breastfeeding, and diet (Figures 2B–2E and S4). To account for the potential sensitivity of beta-diversity metrics to low fungal read counts, we assessed Euclidean distances calculated from centered log-ratio (CLR)-transformed data. Group differences were evaluated using PERMANOVA with sequencing depth included as a covariate, alongside complementary ANOSIM and PERMDISP analyses. Although PERMANOVA indicated statistical significance in some comparisons, ANOSIM did not reveal clear group separation, and PERMDISP was significant in several cases, indicating heterogeneous dispersion among groups. Together, these findings suggest that the observed PERMANOVA significance is more likely attributable to differences in within-group dispersion rather than true shifts in group centroids, thereby limiting the biological interpretability of these results. Consistent with this interpretation, no specific or reproducible clustering of samples was observed in this additional analysis (Figure S5). Therefore, the fungal community structure cannot be explained by these environmental factors and was similar between MZ and DZ twins. It behaved unexpectedly differently from the bacterial community of the same cohort, in which a clear influence of age and household sharing was observed.^28^ Taken together, these data show that the fungal community is far more individualized than the bacterial community, even from the earliest stages of life. While the bacterial community appears to be relatively stable, the gut microbiota show significant individual mycobial variability, as supported by data from the twin cohort assessing genetic and environmental factors.

### Main fungal genera are heterogeneously distributed in a twin cohort

**Figure 3:**
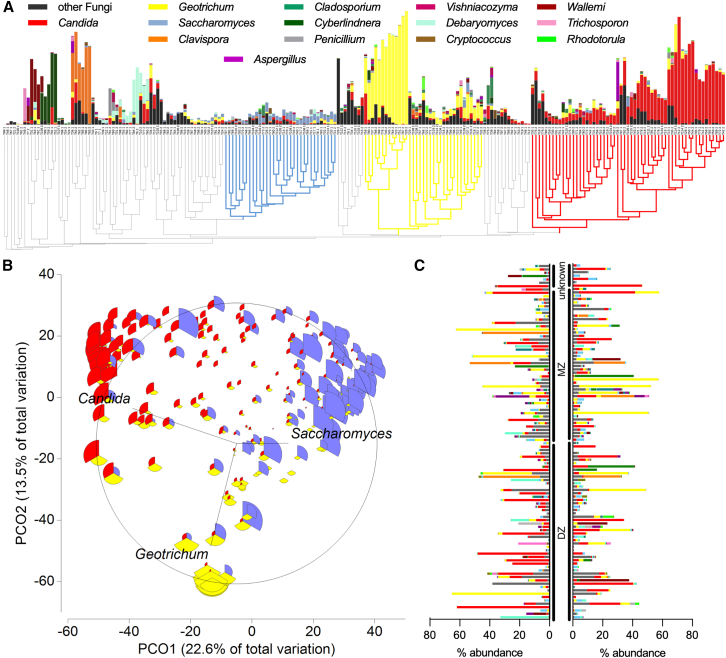
Fungal community in mono- and dizygotic twins (A) Hierarchical clustering of 212 fecal twin samples based on Bray-Curtis resemblance of fungal genera. The relative abundance of all fungal genera, which are present in 10% of the cohort, is displayed, and the sum of other fungi is added. The most representative clusters are marked by color. (B) PCO of the fungal reads of all samples based on Bray-Curtis similarity. Vectors and bubble plots represent the distribution of the most abundant genera *Candida*, *Geotrichum,* and *Saccharomyces* across the sample. (C) Relative abundance of all fungal genera, which are present in 10% of the cohort, is matched for each twin pair, sorted for their zygosity status. MZ: monozygotic, DZ: dizygotic.

Fungal genera abundances were analyzed by again hierarchically clustering twin samples at the genus level, with no twin pairs clustering together. Interestingly, analysis of the gut mycobiome revealed several fungal clusters with high similarity between unrelated samples due to similar fungal abundances, with the three largest clusters belonging to the genera *Candida, Geotrichum,* and *Saccharomyces* (Figure 3A). The abundance of fungal genera was heterogeneous, with a high abundance of *Candida, Geotrichum, Clavispora, Cladosporium,* and *Wallemia* and a low abundance of mainly *Saccharomyces*. The observed variations in abundance can be attributed to the substantial heterogeneity in the total number of fungal sequences obtained (Figure S6). Of all 106 fungal genera (3 phyla), only *Candida* and *Geotrichum* were detected in more than 50% of the samples, followed by *Saccharomyces* (detected in 92 samples), and all other fungal genera were randomly detected in the cohort (including *Cladosporium*, *Penicillium*, *Aspergillus*, Figure 3A). Principal coordinate analysis (PCoA) revealed that *Candida*, *Geotrichum,* and *Saccharomyces* had negative correlations to each other in our cohort (Figure 3B), similar to the results of Nash et al.^19^ The clusters of these three most common genera were tested for association with the aforementioned individual or environmental factors, such as age and diet but again no significant factors were identified. With the exception of the slightly higher number of DZ twins in the *Candida* cluster (Figure S7). The matrix of mycobiome profiles based on zygosity status showed a very heterogeneous picture with no clear correlation (Figure 3C). In addition, no significant differences were observed in the CLR-transformed abundances of *Candida*, *Geotrichum*, or *Saccharomyces* across any of the previously tested groups (Figure S8), strengthening that the relative abundance of these dominant fungal genera was not systematically associated with host characteristics or environmental exposures examined in this study.

### Establishing a model to analyze the merged bacterial and fungal data

To gain a more detailed perspective, we integrated microbiome and mycobiome data to elucidate the interactions between the bacterial and fungal kingdoms and to assess the combined microbial dynamics in twins. To do this, the total bacterial and fungal reads were combined and normalized (Figure S9). To assess whether this merging step had an impact on the data analysis, the fungal-based Bray-Curtis similarity was compared before and after normalization, which showed no statistical difference. Consistent with previously reported bacterial data,^28^ the merged Bray-Curtis similarity of the twin pairs, as expected, was significantly associated with age and household sharing (Figure S9). After systematic evaluation, we confirmed the utility and validity of the implemented model, excluding potential bias due to data merging, and proceeded to the next step of combined data analysis.

### Interindividual variation in the global bacterial and fungal assemblages

In the merged community, species richness, Simpson index, Shannon index and rarity index between a pair of twins were determined by the bacterial community (Figure S10). Thus, species richness in the merged community had an average of 237 per sample, similar to bacterial richness (228), compared to a richness of only 10 in the fungal community, similar to previous results.^17^^,^^19^^,^^22^ While both the Simpson and Shannon indices displayed similar bacterial diversity in all samples, the fungal community showed high variance between samples, suggesting that there are samples with high fungal diversity and others with low fungal diversity.

Among the 138 bacterial genera (12 phyla), *Bacteroides* and *Prevotella* were most frequently detected, followed by *Faecalibacterium, Bifidobacterium, Eubacterium, Alistipes,* and *Ruminococcus*, although many other genera were detected in small amounts in more than 106 samples (50% of the cohort, Figure S11). Overall, the main microbial community of *Candida* and *Geotrichum* and the bacterial genera mentioned above accounted for more than 60% of the merged community in most samples of this cohort. The Bray-Curtis similarity between a pair of twins ranged from 2 to 70%, mostly determined by the shared presence of fungal genera in both twins (Figure S12).

### Bacterial and fungal correlations in the lower GI

**Figure 4:**
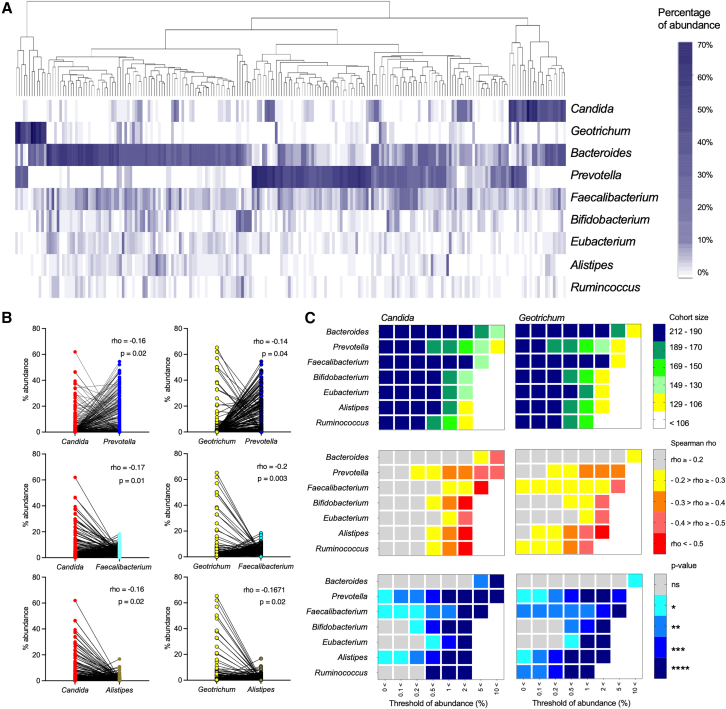
Pairwise correlation analysis between dominant bacteria and fungi (A) Heatmap showing the percentage of abundance of the major microbial community clustered for the Bray-Curtis similarity of the sample. (B) Paired abundances of selected fungal and bacterial genera with significant Spearman’s correlation. Genera abundances in one twin are connected by lines. (C) Heatmap-like plots of *Candida* (left) and *Geotrichum* (right) across each major bacterial genus (y axis) and as a function of threshold % abundance (x axis). The color visualizes the number of samples with co-occurrence of the genus pair, their Spearman’s correlation value rho, and the corrected *p*-value. Significance indicated by: ∗*p* < 0.05, ∗∗*p* < 0.01, ∗∗∗*p* < 0.001, and ∗∗∗∗*p* < 0.0001. All values are shown only when the co-occurrence of the genera was present in 106 samples (50% of the cohort).

The main microbial community revealed a heterogeneous abundance profile, as *Candida* was detected in 156 samples with a relative abundance >0.1% but only in 80 samples with >2%, while *Geotrichum* was detected in 122 samples and in 49 samples at those relative abundances. The same can be said regarding the bacterial taxa (*Bacteroides:* 209 and 192 samples, *Bifidobacterium:* 194 and 84, at those abundances, Figure 4A). These abundance profiles revealed specific co-occurrence patterns: Both raw and CLR-transformed data often showed that bacterial genera were absent when fungal genera were highly abundant (Figures 4A and S13), resulting in a significant negative correlation (Figure 4B). These co-occurrences were further analyzed as pairwise taxa correlations depending on the abundance thresholds, calculated only when one of the two correlated taxa was present in at least 50% of the cohort (Figure 4C). Almost all bacteria showed negative correlations with *Candida* above an individual abundance threshold, which became stronger with increasing genus abundance (Figures 4C and S14). To illustrate, *Bacteroides* did not show significant negative correlation with *Candida* when its abundance was below 5%, while *Prevotella, Alistipes,* and *Faecalibacterium* showed negative correlation against this fungus already at an abundance of 0.1% (Figures 4B and 4C). Considering the main community members in our cohort, the genera *Alistipes*, *Bifidobacterium*, and *Ruminococcus* showed a strong negative correlation with *Candida* (Spearman's rank correlation coefficient of −0.6 with a corrected *p* value < 0.0001) when their relative abundances were ≥2%. The same can also be said for *Oscillibacter* and *Parabacteroides* (Figures 4C and S14). Similar negative correlation patterns were detected when *Geotrichum* was correlated; however, *Geotrichum* was present in much lower relative abundances than *Candida*, decreasing the number of ranges in which the correlations were calculated, especially when bacteria in low abundances were considered (Figure S14). Negative correlations between bacterial taxa and fungi have been previously suggested^17^^,^^18^^,^^19^^,^^20^^,^^21^^,^^22^ but never linked to the relative abundance. To confirm our model, we utilized publicly available data,^17^ which show a similar pattern and support our methodology and conclusions (Figure S15).

### Unraveling the interkingdom communities’ network in the lower GI

**Figure 5:**
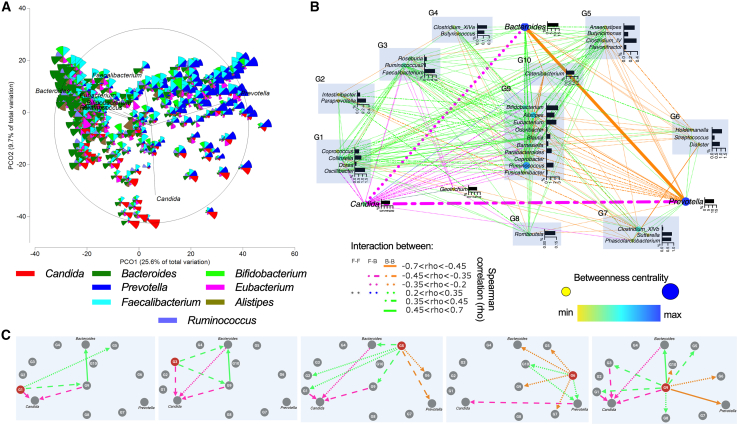
Negative interkingdom relationships shaping the merged fungal and bacterial community (A) PCO analysis of all genera present in 50% of the cohort based on Bray-Curtis similarity. Vectors and bubble plots of the major bacterial genera and *Candida* represent the distribution of genera. (B) Iterative community network with the average abundance for each genus shown. Nodes represent betweenness centrality and are grouped by their interactions with the three most abundant taxa (*Bacteroides, Prevotella*, and *Candida*). All genera present in 106 individuals (50% of the cohort) were considered. Interactions show Spearman correlation (0.2 ≤ rho ≥0.2, corrected *p*-value<0.05). (C) Summary of consistent network interactions between bacterial groups and the three most abundant taxa.

Having shown the negative correlation between the most abundant fungal and bacterial genera, we aimed to further investigate the cross-kingdom interactions embedded in a community. Negative interactions between taxa were apparent in the PCoA of the main microbial community (Figure 5A) and, notably, remained consistent after the CLR transformation of abundance data, indicating that these interaction patterns are not driven by data scale or compositional effects (Figure S16). *Candida, Prevotella*, and Bacteroides dominated the microbiome in most samples, with a clear opposing relationship between them, while the other genera shown were only associated with *Bacteroides*. Those findings were statistically supported by the community network, displaying most of the described interactions (Figure S17). To visualize the correlations detected in Figure 4C, an iterative network was created by merging 6 subgroup networks, which revealed all the measured correlations (Figure 5B, merging shown in Figure S18). All 37 genera within the community had 10 different types of interactions, labeled as bacteria groups G1 to G10, depending on their interactions with *Candida, Prevotella,* and *Bacteroides* (the most abundant genera, Figure 5B). Overall, *Bacteroides* and *Prevotella* showed a strong negative interaction toward each other again (Figures 5A–5C). However, G9 was a key group consisting of ten bacterial genera, with *Bifidobacterium*, *Alistipes*, *Eubacterium,* and *Ruminococcus* being the most abundant. This group has strong positive interactions with *Bacteroides* and negative interactions with *Prevotella*, as well as with *Candida*. In addition, groups G1 and G3 are in direct negative interaction with *Candida*. *Prevotella* also showed a strong negative interaction with *Candida* without belonging to any bacterial group (Figure 5C). The iterative community network revealed various negative interactions between the bacterial community and *Candida*. Supported by the results of Figures 4A, 5A, S16, and S17, it can be hypothesized that such negative interactions may lead to a protective function of the different bacterial colonizations against *Candida*.

## Discussion

Analysis of the microbiome in twins has contributed to a better understanding of bacterial^23^^,^^24^^,^^25^^,^^26^^,^^27^^,^^28^ and viral^29^ communities. Despite the increasing appreciation of fungi in health and disease,^4^ data on the dynamics of the mycobiome, in particular its cross-kingdom interaction, remain limited. In this work, we attempted to gain a comprehensive view of the mycobial and microbial composition in the gut of MZ and DZ twins by exploring their interplay, taking advantage of controlled genetic and environmental factors. Overall, our analyses indicate that the human mycobiome in this cohort is characterized by pronounced inter-individual variability and a limited degree of structured association with host, environmental, or dietary factors. Despite extensive testing across demographic variables, early-life exposures, nutritional intake, and zygosity, neither global fungal community structure nor the relative abundances of the most prevalent genera (*Candida*, *Geotrichum*, and *Saccharomyces*) showed consistent or biologically meaningful associations. Importantly, these conclusions were robust to methodological considerations, including low fungal read counts and compositionality, as demonstrated by consistent results obtained using CLR-transformed data and multiple beta-diversity frameworks. In contrast, negative interactions between taxa within the broader microbial community were reproducibly observed and remained stable following CLR transformation, supporting the notion that these interaction patterns represent robust ecological relationships rather than artifacts of sequencing depth or data scaling. Together, these findings suggest that while fungal community composition is highly variable, stable inter-taxa interactions may represent a more consistent feature of the microbial ecosystem than associations with host or environmental covariates.

The data in this paper show no statistical correlation between the fungal community and various factors such as zygosity status or twin age. This is in clear contrast to the results of the work of Vilchez-Vargas et al., in which the bacterial community of the same twin cohort was analyzed.^28^ There, too, the influence of genetics was limited, but the bacterial community was highly influenced by the age and cohabitation status of the twins. Specifically, this meant that young twin pairs had a more similar microbiome than older ones, which is probably due to the fact that they usually live together in the same household and share the same environment and diet.^28^ The data from Moreno-Gallego et al. also show that viruses follow the same trend as bacteria and that the viral community of twins diverges over the course of life.^29^ However, our present data show that this is not the case for fungi. It may be hypothesized that the bacterial and viral communities are more closely and positively linked than the bacterial and fungal communities, possibly because most viruses in the microbiota are bacteriophages that coexist simultaneously with bacteria.^29^

Most members of the fungal community detected in the present twin cohort are quite common in the human lower GI. Different studies have already found especially Candida and Saccharomyces, but also Geotrichum, Pichia, Clavispora, and others.^17^^,^^19^^,^^20^^,^^21^^,^^22^^,^^30^^,^^31^ We revealed that different individual twins were mostly colonized by one dominant fungus, resulting in a very clear clustering of the most common fungi *Candida*, *Geotrichum,* and *Saccharomyces*. We can assume an antagonistic relationship between the different fungal genera, as other studies have also found negative correlations between them,^19^ but this negative correlation has not been investigated mechanistically, and other environmental factors that shape the microbiota could also have an influence on the fungal community. Our cohort showed a notably low abundance of *Saccharomyces* compared to previous studies.^19^ To ensure data quality, we carefully controlled for potential contamination by including negative controls during PCR, and we verified taxonomic annotation using both the UNITE database^32^ and the BLAST algorithm against the NCBI database.^33^^,^^34^ Although we did not identify any technical or biological factors within our dataset that could account for this discrepancy, we cannot exclude the possibility of regional differences in the gut mycobiome composition specific to the Lithuanian population or other unmeasured environmental or dietary influences.

Diet is one factor that has been extensively studied for its influence on the microbiota, and there are also data highlighting the importance of diet for the fungal community. David et al. showed that the short-term consumption of diets consisting entirely of animal or plant products altered the microbial community structure and that food-borne fungi from both diets transiently colonized the gut.^35^ Despite these data, we cannot associate the dietary intake of the individuals studied with a specific fungal profile, but this cohort was not designed to do so. We acknowledge that the cross-sectional design of our study provides only a snapshot in time and does not capture temporal dynamics or potential seasonal influences on the gut mycobiome. Additionally, some detected fungi may represent transient organisms rather than stable gut colonizers, particularly taxa observed sporadically across the cohort, such as *Clavispora*, *Wallemia*, and *Debaryomyces*. Whether these transient fungi are food-borne or originate from other environmental sources could not be determined in this study.

The relationship between the microbiota and age has been extensively discussed in the literature, with data also emerging on fungi. Lai et al. reviewed several datasets on the fungal colonization of the human GI tract and found that *Candida* abundance is higher with age and confers an increased risk of several diseases associated with a compromised intestinal barrier.^31^ Martino et al. also found that certain fungal genera are associated with specific stages of life, for example, in the first days of life, the fungal community is dominated by Rhodotorula and Debaryomyces spp, followed in the next month by Candida, Cryptococcus, and *Saccharomyces*. In adulthood, the dominant fungal genera remain *Aspergillus*, *Candida,* and *Saccharomyces.*^36^ Despite the wide age range of our cohort, we could not confirm such an association of certain fungal genera with age, which shows the complexity of this research question and the further amount of data needed to solve it.

In addition to age and diet, other environmental factors may influence the gut mycobiome. However, the complexity and diversity of environmental exposures are difficult to comprehensively capture and analyze. Although we attempted to account for shared environmental influences by assessing cohabitation status, this approach can only partially address environmental variability. Consequently, the generalizability of our findings to broader populations remains limited.

Taken together, one of the most striking observations of this study is the high degree of inter-individual variability in gut fungal composition. While our analyses suggest that host genetics, age, diet, cohabitation, and other major environmental factors assessed here do not explain a substantial proportion of this variation, the drivers underlying individual-specific mycobiome profiles remain unclear. This variability may arise from a combination of unmeasured influences, including early-life exposures, transient ingestion of food-borne or environmental fungi, host immune factors, stochastic colonization events, and short-term environmental fluctuations. In addition, technical factors inherent to low-biomass fungal sequencing may contribute to variability and cannot be fully excluded. Together, these considerations indicate that the gut mycobiome may be shaped by highly dynamic and individualized processes rather than stable, host-determined factors. Elucidating the mechanisms governing this variability will require longitudinal sampling, improved quantification of environmental exposures, and integrative multi-omics approaches that capture functional and metabolic activity. While beyond the scope of the present study, our findings highlight this unexplained variability as a key challenge and an important direction for future research in human mycobiome biology.

For further analysis, the bacterial and fungal communities were combined by resampling the data together. In the literature, fungal communities are usually analyzed in the same way as bacterial communities. For example, Lemoinne et al.^22^ only considered the samples with ≥2,900 fungal reads and Nash et al.^19^ with ≥1,954 fungal reads, removing the samples below these cut-offs. With a cut-off of 2,900 reads in our cohort, 183 samples would have been removed; with a cut-off of 1,954 reads, 165 samples would have been removed, so that a significant number of samples would not have been considered for analysis. However, these cut-offs were chosen for technical reasons only because the number of reads appeared to be too small to be sufficiently representative. Our results suggest that while this approach works well for bacteria, it is not suitable for fungi. We believe it is important to consider samples with a low sequence count, as this low count may be due to biological reasons in fungi and is not just a technical. Since all negative controls during the PCR procedure remained negative, it can be assumed that the fungi detected are colonizers of the gut, even in low abundance, and not contaminants. By removing these samples, important information is lost, as fungi, unlike bacteria, are not universally present in the lower GI, but only occur in certain individuals. Our results show that the causes of fungal colonization may also lie in the bacterial community, as these show clear negative correlations with fungi. In order to better understand these relationships, we have developed a strategy in which all samples are considered for analysis. In this approach, we are aware that we are assuming the same number of bacteria in all fecal samples, in contrast to other studies reported previously, where no normalization was performed.^17^ Indeed, our analysis also assumes comparable biomass contributions from bacteria and fungi. Although fungi are relatively rare in the gut microbiome, their substantially larger size suggests they contribute a meaningful proportion of the total intestinal microbiome biomass.^6^

With our merged community analysis strategy, we have made great efforts to analyze the relationships between fungi and bacteria. We found convincing results, showing strong negative correlations between members of the main microbial community and the fungi *Candida* and *Geotrichum*, as a function of relative abundance. Increasing the abundance threshold also adjusts for any bias potentially introduced by sequences with very low abundance. Furthermore, we were able to validate our approach using publicly available data from Hoffman et al.^17^ By following the exact procedure for resampling the data and performing the correlations, we found the same pattern of negative correlation between different bacteria and *Candida*. For example, *Alistipes*, *Faecalibacterium*, *Ruminococcus,* and *Parabacteroides* had negative correlations to *Candida,* and *Candida* also to *Saccharomyces*. *Bacteroides* was much more abundant in the Hoffmann et al.^17^ cohort than in our cohort (>10% in 86 of 91 samples). Consequently, the negative correlation with the relative abundance of *Candida* was stronger.

The opportunistic pathogen *Candida* was detected in our cohort regardless of age and diet, and *Prevotella* appeared to have the strongest negative correlation with it, leading to the initial conclusion that high abundances of *Prevotella* naturally protect against *Candida* infections. Interestingly, *Prevotella* was not only negatively correlated to *Candida*, but also to almost all bacteria in the human lower digestive tract, including *Bacteroides*, *Alistipes*, *Eubacteria,* and *Ruminicoccus*. The negative correlation of *Bacteroides* and *Prevotella* proved to be an important component of the community network and has been reported previously.^37^ Furthermore, *Bacteroides* was fully integrated into the community, similar to the findings of Levy et al.^38^ The bacteria associated with *Bacteroides* were all negatively correlated with *Candida*. Overall, this leads to the hypothesis that the bacterial community may have two ways of resisting colonization by *Candida*, either by a high abundance of *Prevotella* or by *Bacteroides* and its associated bacteria.

In our study, we can only describe associative patterns, and the mechanisms underlying our hypothesis in the human gut remain to be elucidated. However, the pronounced negative correlations between specific bacterial genera (e.g., *Alistipes*) and fungal taxa (e.g., *Candida* and *Geotrichum*) may suggest a model of competitive exclusion. This might be driven by bacterial metabolite production, such as short-chain fatty acids, which are known to modulate fungal morphology and colonization resistance.^39^ Also, further mechanistic evidence from other models already indicates that bacteria can protect the host against *Candida* infection. Authier et al.^15^ performed experiments with the oral administration of *Lactobacillus* in a mouse model and were able to alleviate gastrointestinal candidiasis. Other studies have found that *Streptococcus mutans* can prevent *Candida* from adhering to epithelia in the human body and thus prevent it from becoming pathogenic.^16^ *Bacteroides thetaiotaomicron* can also process the cell wall of *C. albicans* via a mannose pathway.^40^ However, these bacteria were not common lower GI colonizers in our cohort. Nevertheless, they support our hypothesis that the colonization of the human lower GI with certain bacteria could prevent infection with certain *Candida* species. This antagonistic relationship could be exploited in medicine to shape the bacterial community, either by fecal microbiome transplantation or by utilizing probiotics to construct a microbiome that could prevent fungal colonization. To the best of our knowledge, there has been no application of the bacteria found in our study as probiotics, and certainly not to specifically prevent fungal colonization. Further studies are needed to address this in the future.

Nevertheless, it is important to acknowledge that, while our study provides a unique and informative dataset, it also has certain limitations. First, both 16S rRNA gene and ITS sequencing are inherently subject to biases introduced by PCR-based amplification; therefore, future studies—particularly those investigating cross-kingdom interactions—would benefit from PCR-independent sequencing approaches. Although our analysis establishes a robust landscape of fungal–bacterial antagonism within a twin cohort, the molecular mechanisms underlying these associations remain to be clarified. Future work should prioritize longitudinal, multi-omic profiling, integrating metatranscriptomics and metabolomics, to determine whether the observed negative correlations, such as those between *Alistipes* and *Candida*, are driven by specific antifungal metabolites (e.g., short-chain fatty acids or secondary bile acids) or by competition for shared nutrient niches. In addition, experimental validation using *in vitro* synthetic microbial communities could help establish whether these bacterial genera actively confer colonization resistance against fungal overgrowth. From a clinical perspective, a deeper understanding of cross-kingdom microbial interactions may enable the development of “mycobiome-aware” probiotics, in which bacterial strains are selected not only for their individual benefits but also for their capacity to maintain fungal homeostasis.

In conclusion, detailed analysis of the mycobial community of twins strongly suggests that the fungi are independent of various personal and environmental influences and do not play an important role in determining microbial similarity in twins. We also show strong interactions between the bacterial and fungal communities, and the detected negative correlations seem to be a keystone between these communities. Further investigation is warranted, as these correlations may present a potential for interference with the bacterial community, thereby influencing its composition and potentially preventing fungal colonization.

### Limitations of the study

We acknowledge the moderate sample size of our cohort. However, given the constraints of maintaining an environmentally and genetically controlled group and the relatively small population of Lithuania, increasing the cohort size was not feasible at this stage. Importantly, the use of a human twin study model with a uniquely broad age distribution, combined with adjustment for numerous influencing factors, ensures that even a smaller, targeted cohort can yield scientifically robust and valuable findings. This approach offers a distinct perspective on fungal–bacterial interactions, minimizes confounding factors, and adds another direction to the existing understanding. Nevertheless, we recognize that the findings may be region-specific and cannot be directly generalized to other populations.

## Resource availability

### Lead contact

Requests for information and resources should be directed to and will be fulfilled by the lead contact: Alexander Link (alexander.link@fau.de).

### Materials availability

Any unique materials generated in this study are available upon request from the lead contact.

### Data and code availability

• Raw 16S rRNA and ITS sequencing data are available from the National Center for Biotechnology Information Sequence Read Archive with the accession number BioProject: PRJNA1254145.
• This paper does not report original code.
• Any additional information required to reanalyze the data reported in this paper is available from the lead contact upon request.

## Acknowledgments

We would like to thank Mrs. Ilka Kramer for excellent technical assistance. Furthermore, we thank the team at the Data Integration Center of University Medicine Magdeburg for local data-analysis solutions, supported by MIRACUM and funded by the German Federal Ministry of Education and Research (10.13039/501100002347BMBF) within the “Medical Informatics Funding Scheme” (FKZ 01ZZ1801H). We would like to thank Mrs. Erin Calabria for editing and proofreading. This work was supported by the European funds for regional development under grant ID: ZS/2018/11/95324; 10.13039/501100002347German Federal Ministry of Education and Research under grant ID: 01GM2204A; and the 10.13039/501100004504Research Council of Lithuania under grant no. S-MIP-23-101. We acknowledge support by the Open Access Publication Fund of Magdeburg University.

## Author contributions

Formal analysis: K.L., J.S., N.M.H., and RVV; visualization: K.L., C.T. and RVV; conceptionalisation: R.V.V., J.K., and A.L.; software: R.V.V.; methodology: R.V.V.; data curation: J.S., G.G., I.G., L.K. and M.U.; resources: J.S., G.G., I.G., L.K. M.U., C.T., D.S., M.Z., and J.K.; funding acquisition: A.L.; project administration: A.L.; writing (original draft): K.L., R.V.V., and A.L.; writing (review and editing): all authors.

## Declaration of interests

A.L. received a speaker or advisory fee from Janssen, Ferring, and Luvos. All other authors declare no conflict of interest.

## Declaration of generative AI and AI-assisted technologies in the writing process

During the preparation of this work, the author(s) used ChatGPT and DeepL Write in order to check for spelling mistakes and improve overall readability. After using this tool or service, the author(s) reviewed and edited the content as needed and take(s) full responsibility for the content of the publication.

## STAR★Methods

### Key resources table

**Table:** 

REAGENT or RESOURCE	SOURCE	IDENTIFIER
**Biological samples**		

Fecal sample from healthy twins	Twin Registry Center at Lithuanian University of Health Sciences	P1-52/2005

**Chemicals, peptides, and recombinant proteins**		

QIAamp Fast DNA Stool Mini Kit	Qiagen	CAT# 51604

**Deposited data**		

16S rRNA gene Data	This Paper	BioProject: PRJNA1254145
ITS Data	This Paper	BioProject: PRJNA1254145

**Software and algorithms**		

R Statistical Software version 4.2.1	R Foundation for Statistical Computing	https://www.r-project.org/
Prism 7	GraphPad Software	https://www.graphpad.com/
Primer 7 with PERMANOVA+ add-on package	PRIMER-E	https://www.primer-e.com/
Cytoscape version 3.8.0	Cytoscape	https://cytoscape.org/

### Experimental model and study participant details

The twins were included in the study from the Twin Registry Center at Lithuanian University of Health Sciences (years 2016–2018). The study included 106 twin pairs with 45 MZ, 54 DZ and 7 twin pairs with unknown zygosity status (212 individuals, Figure S1), which was genetically confirmed.^28^ The individuals were further allocated in groups for comparison by cohabitation status, gender, brest feeding status and way of birth (Figure S1). The study was performed in accordance with current good clinical practice guidelines and was approved by the local ethics committee (Protocol No: P1-52/2005). All individuals gave their written informed consent prior to inclusion in the study. All subjects were in good health and have not been taking any probiotics, antibiotics or antifungal drugs before sampling.

Individuals delivered one fresh fecal sample each which was stored at −80°C until DNA extraction. Procedure for zygosity determination and nutrition intake calculation are previously described in detail.^28^ Briefly, zygosity testing (MZ versus DZ) was performed on blood DNA samples. Short tandem repeat polymorphic DNA markers were amplified by PCR using AmpFLSTRÒ IdentifilerÒ Plus PCR Amplification Kit (Thermo Fisher Scientific, USA).

### Method details

DNA was extracted from all 212 stool samples using QIAamp Fast DNA Stool Mini Kit (Qiagen, Venlo, Netherlands). The extracted DNA was sent to the Otto-von-Guericke University in Magdeburg (Germany) for further analysis. To analyze the bacterial community, amplicon libraries were generated by amplifying the V1-V2 region of the 16S rRNA gene after 20 cycles PCR reaction using the 27F and 338R primers.^41^ The data on bacterial communities of this cohort were previously reported.^28^ The fungal community was characterized amplifying a fragment from the ITS1-ITS2 region with the primers ITS1f^42^ and ITS2r,^43^ after 40 cycles PCR reaction. The sequences of the fungal primers are available in the earth microbiome project^44^ and they were previously reported as the potentially best currently reported primers for analyzing the fungal community.^45^ At all PCR steps a negative control was introduced to detect contamination.The same extracted DNA was used, and the sequencing was performed on the same Illumina MiSeq (2 × 250 bp, Illumina, San Diego, USA).^46^

#### Quantification and statiastical analysis

The initial bacterial (phylotypes) and fungal (mycotypes) sequence tables were generated from fastQ files after sequencing and demultiplexing, in R Statistical Software version 4.2.1 (2020; R Foundation for Statistical Computing, Vienna, Austria) using the dada2 package version 1.10.1.^47^ The phylotypes were annotated with the Ribosomal Data Project (version 2.13)^48^ and the mycotypes with the UNITE database (version 8.2).^32^ The most abundant sequences were manually verified with the Basic Local Alignment Search Tool against the NCBI nucleotide database.^33^^,^^34^ A genus name was assigned to a sequence based on the naive Bayesian classification^49^ with a pseudo-bootstrap threshold of 80%.

The resampling was done to analyze both communities coexisting in the same set of samples. First, Good’s coverage estimator^50^ was calculated in percentage, as the ratio of the number of singletons and the sum of all phylotypes per sample. The sequencing depth for the bacterial phylotype table was fixed to the previous study,^28^ at 4,838 reads in order to reach an optimal Good's coverage (Good’s coverage = 99.5%, Figure S19) and for easier comparison. The normalized phylotype table was merged with the mycotype table of the fungal sequences, and the resulting table was resampled to the minimum number of 4,842 sequences. For all resampling steps the R-package phyloseq^51^ was used. As a consequence, all samples were considered, and further analyses were performed with 631 mycotypes and 9,879 phylotypes, expressed as percentage (Table S1).

The graphical abstract was created in BioRender (Thon, C. 2026 https://BioRender.com/mukqeg2). Further figures were visulisted with using Prism 7 (GraphPad Software, La Jolla, CA) together with applying the Mann-Whitney- and Kruskal-Wallis-test to evaluate the significant differences between *a priori* defined groups. Principal Coordinates Analysis (PCoA) were built using the Bray-Curtis similarity (1 minus Bray-curtis distance) matrix algorithm in Primer 7 with PERMANOVA+ add-on package (PRIMER-E, Auckland, New Zealand). Diversity index of each sample, rarefaction curves and Spearman correlation coefficients were calculated in R using the packages vegan (version 2.6.4),^52^ psych (version 2.3.3)^53^ and reshape2 (version 1.4.4),^54^ respectively. Dendrograms were calculated with Bray-Curtis-similarity in R using the ecodist (version 2.0.9)^55^ and cluster (version 2.1.3)^56^ package. All trees were visualized with the interactive Tree of Life, iToL.^57^ All *p* values in this work were corrected by applying the Benjamini-Hochberg false discovery rate correction.^58^ The applied significance level nomenclature for all tests is: ∗*p* < 0.05, ∗∗*p* < 0.01, ∗∗∗*p* < 0.001, ∗∗∗∗*p* < 0.0001.

Spearman pairwise correlation analyses were performed between the most abundant bacterial and fungal genera (present in 50% of the cohort). Eight sample subgroups were defined according to the relative abundances of the correlated genera. The first subgroup contained only the samples where one of the two correlated genera were >0% abundant, removing those samples where both genera were absent. The second subgroup contained only those samples where one of the two correlated genera had an abundance threshold >0.1%, and successively six groups were defined for abundance thresholds >0.2%, >0.5%, >1%, >2%, >5% and >10%, respectively. Afterward, Spearman tests were performed in an iterative manner for each pairwise comparison in each of the eight groups, but only if the subgroup contained at least 106 samples (50% of the cohort). In light of these considerations, an approach was devised, entailing the calculation of 205 or 170 correlations between the relative abundances of *Candida* or *Geotrichum* and the relative abundances of bacterial genera.

Community networks were generated using Cytoscape^59^ (version 3.8.0) with interactions based on the calculated Spearman coefficients (rho) and displayed only if - 0.2 ≥ rho ≥0.2 and if the corrected *p* value <0.05. The network of the whole community was built with all samples of the cohort. The iterative network was built using the function “merge” in Cytoscape. A total of six networks were merged considering a subgroup of samples at the described abundance threshold >1% of *Candida* and the most abundant bacterial genera, showing a negative correlation between them (Figure S18).

## Supplemental information

Document S1. Figures S1–S19

Table S1. Excel file with the abundances (in %) of the sequences detected in each sample and the DNA sequence of each phylotype and mycotype
