# Supplementary material for: The gut mycobiome is shaped by interactions with the bacterial community in twins
Source: iScience. 2026 Apr 16;29(6):115786. doi: 10.1016/j.isci.2026.115786 (PMC13202532; doi:10.1016/j.isci.2026.115786)
Supplement: Document S1. Figures S1–S19 [file mmc1.pdf]

iScience, Volume *29*

Supplemental information

The gut mycobiome is shaped by interactions with the bacterial community in twins

Konrad Lehr, Ramiro Vilchez-Vargas, Jurgita Skieceviciene, Noam Mathias Hipler, Greta Gedgaudienė, Indre Gustaityte, Laimutis Kucinskas, Mindaugas Urba, Cosima Thon, Denny Schanze, Martin Zenker, Juozas Kupcinskas, and Alexander Link

Age		9 to 72 years<br>Median = 21
Cohabitation	Cohabitated twin pairs	53
	Not cohabitated twin pairs	51
	Unknown twin pair status	2
Gender	Individuals Male	74
	Individuals Female	138
	Twin pairs different gender	28
	Twin pairs same gender	78
Zygosity	Monozygotic twin pairs	45
	Dizygotic twin pairs	54
	Unknown twin pair status	7
Breast feeding	Twin pair with no breast feeding	10
	Twin pair with breast feeding	44
	Unknown twin pair status	52
Way of birth	Twin pair with natural birth	74
	Twin pair with CS	32

Supplementary Figure 1: Characterization of twin pairs.

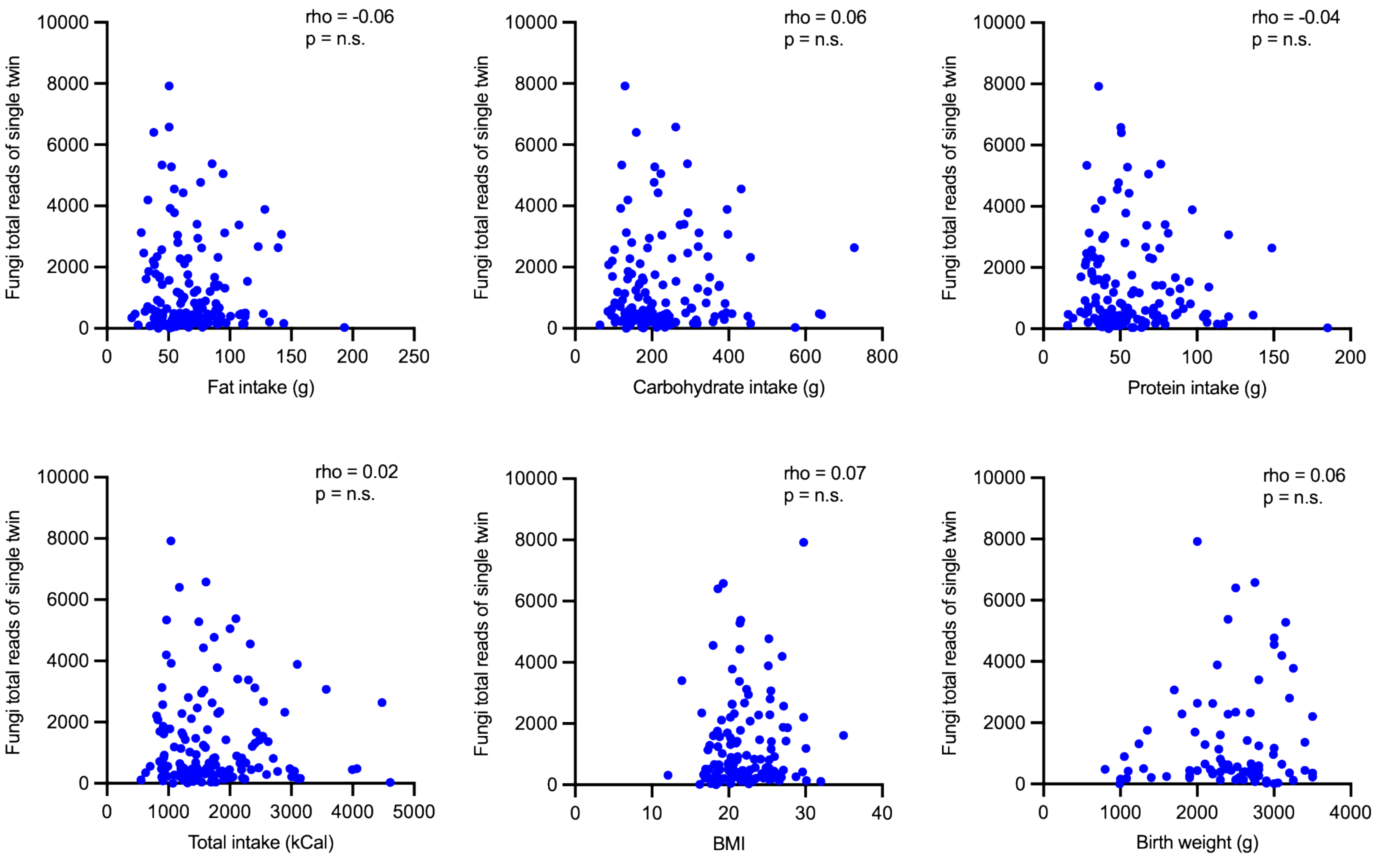

Supplementary Figure 2: Spearman correlation between the total reads of fungi with total intake of fat, carbohydrate, protein or kCal, BMI, weight at birth. None statistical test reached significance level.

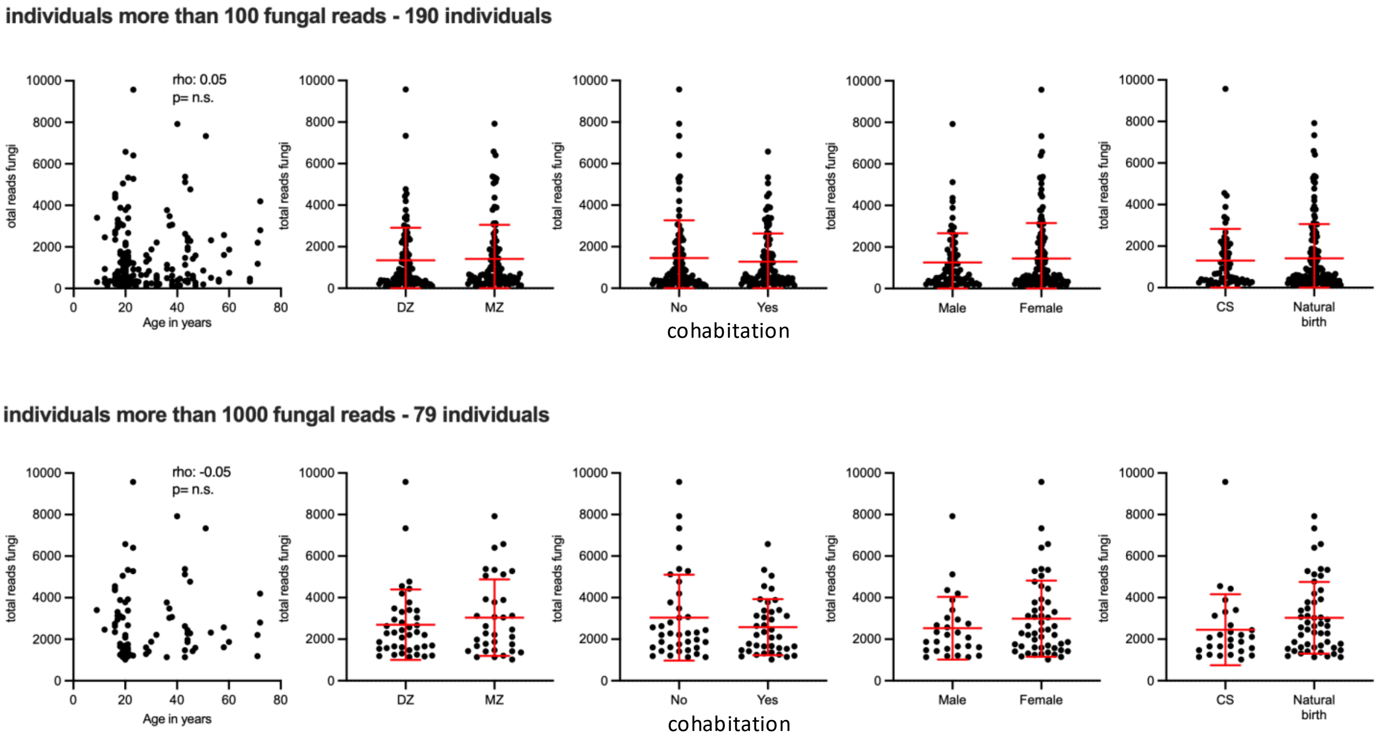

Supplementary Figure 3: Association between age and the sum of fungal sequences in a single twin with Spearman correlation for testing significands level. Zygocity status, houseshared status, gender and way of birth tested for differences in the sum of fungal sequences in a single twin with Mann-Whitney Test. Means +/- standard deviation are displayed. First raw with a cutoff of 100 reads, second raw with a cutoff of 1000 reads. None statistical test reached significance level. MZ: monozygotic, DZ: dizygotic, CS: cesarian section.

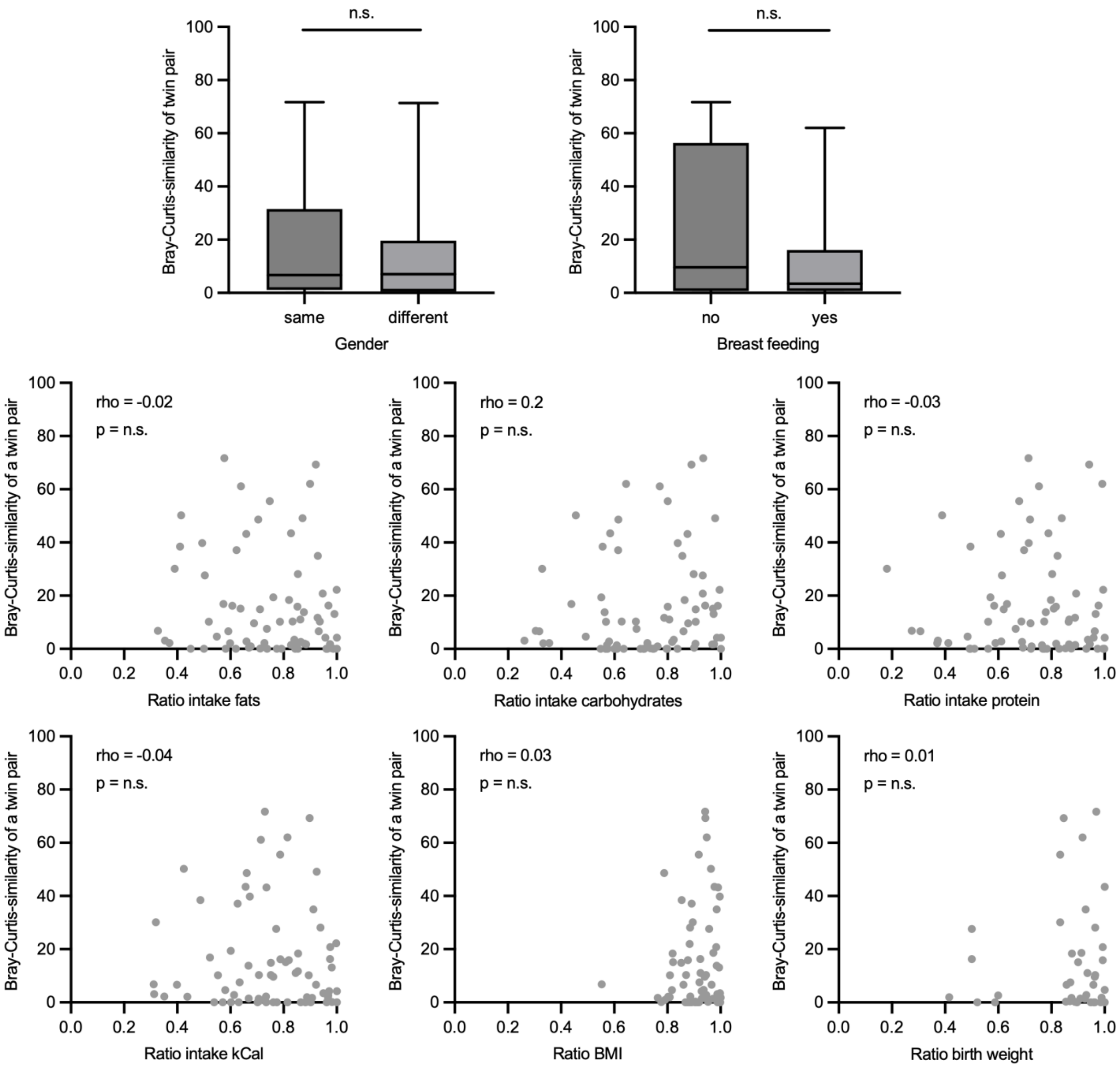

Supplementary Figure 4: Association between the Bray-Curtis-similarity of fungi with gender, breast feeding with Mann-Whitney-Test and total intake of fat, carbohydrate, protein or kCal, BMI, weight at birth with Spearman correlation for testing statistical significance. None statistical test reached significance level. Box plot data are represented as mean +/- standard deviation.

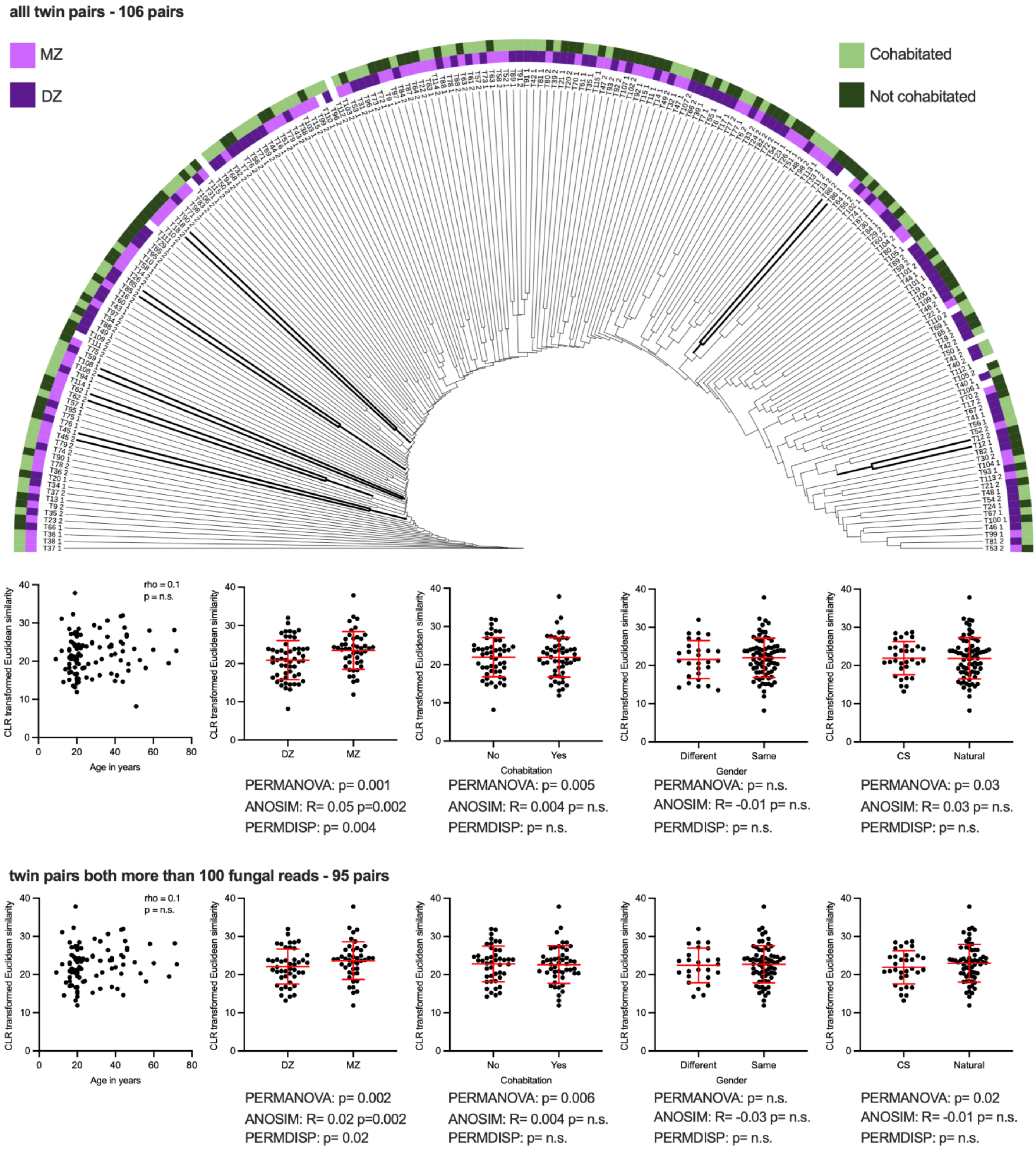

Supplementary Figure 5: Hierarchical clustering of 212 fecal twin samples based on CLR transformation and Euclidian resemblance of fungal reads. Cohabitation and zygosity status are indicated. Spearman correlation analysis between the Euclidian resemblance of fungal reads and the age of the twins. Comparison of the Euclidian similarities of the fungal reads in twin pairs between zygosity status, houseshared status and way of birth. PERMANOVA, ANOSIM and PERMDISP were performed between all groups. Means +/- standard deviation are displayed. First raw with all twins, second raw with a cutoff of 100 reads in both twins. MZ: monozygotic, DZ: dizygotic CS: cesarian section.

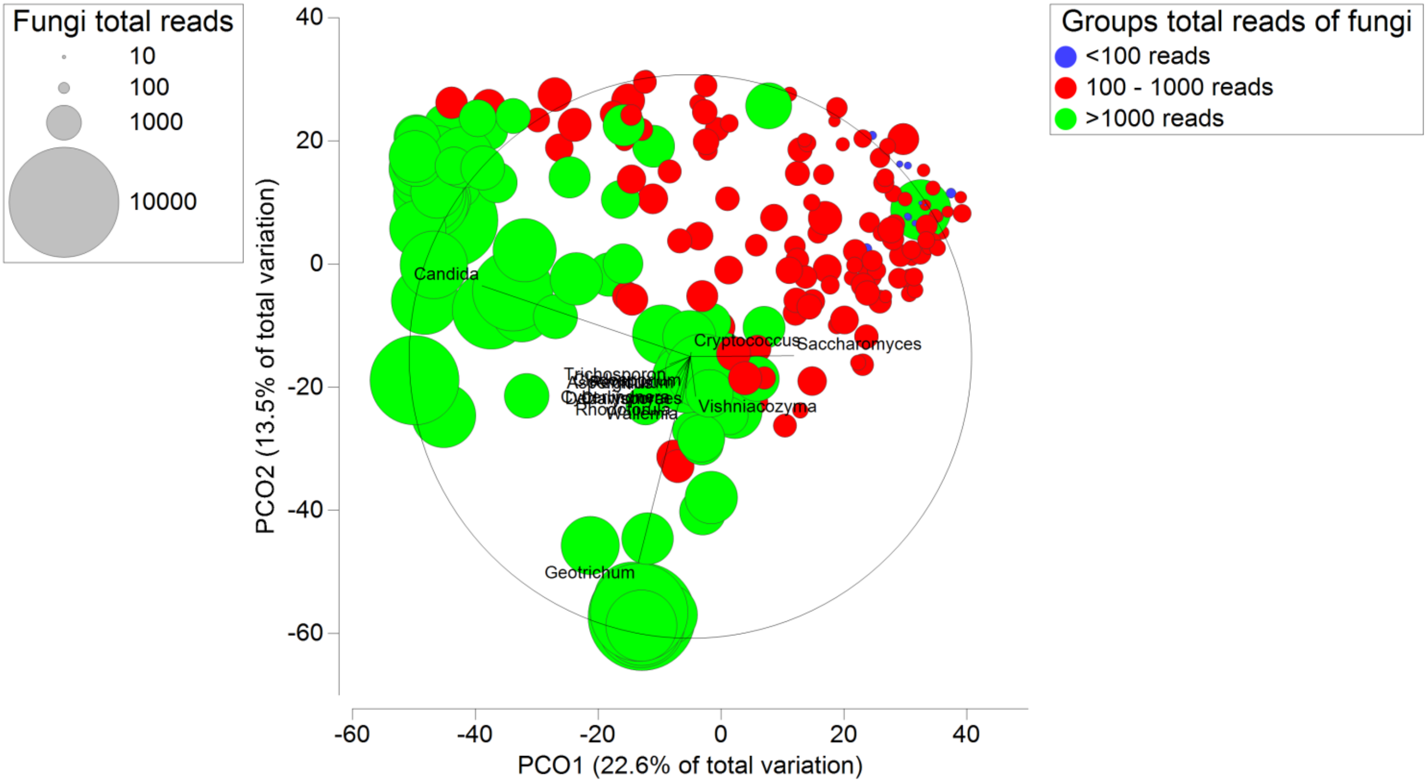

Supplementary Figure 6: PCO of the fungal reads of all samples based on Bray-Curtis similarity. Vectors represent the most abundant Fungi. Bubble plots represent the distribution of total reads of fungi or sequencing depth of fungi across the sample.

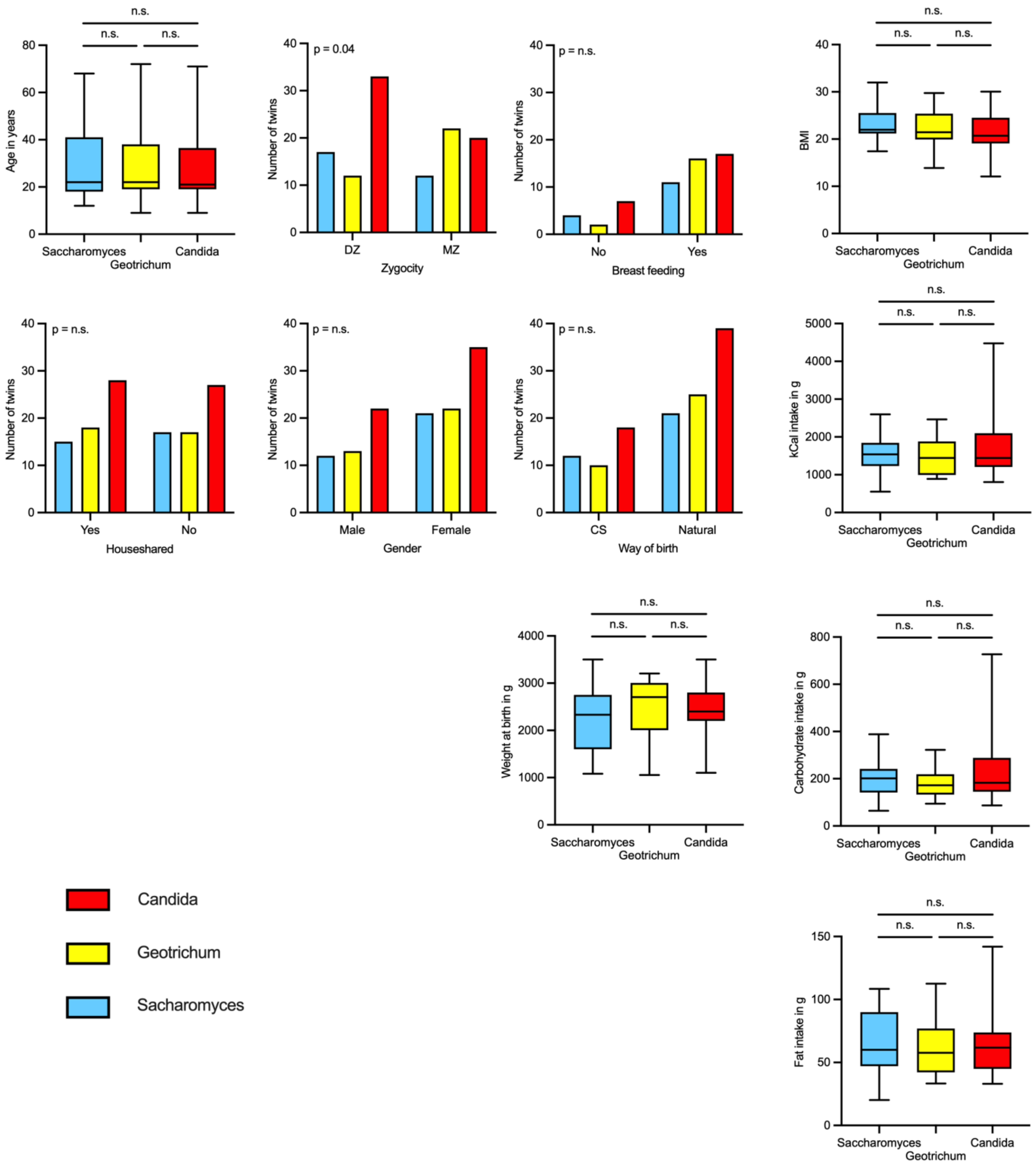

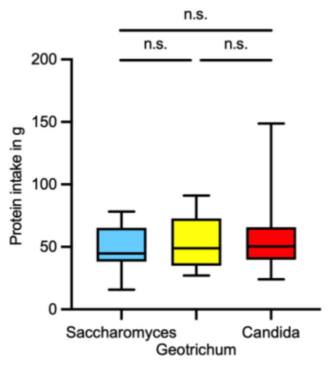

Supplementary Figure 7: Associations between the relative abundance of *Candida, Geotrichum, Saccharomyces* with age, house sharing, zygosity, gender, breast feeding, way of birth, weight at birth, BMI and the intake of fats, proteins, carbohydrates and kCals. Kruskal-Wallis Test with subsequent Mann-Whitney-Test were used for testing statistical significance. Box plot data are represented as mean +/- standard deviation.

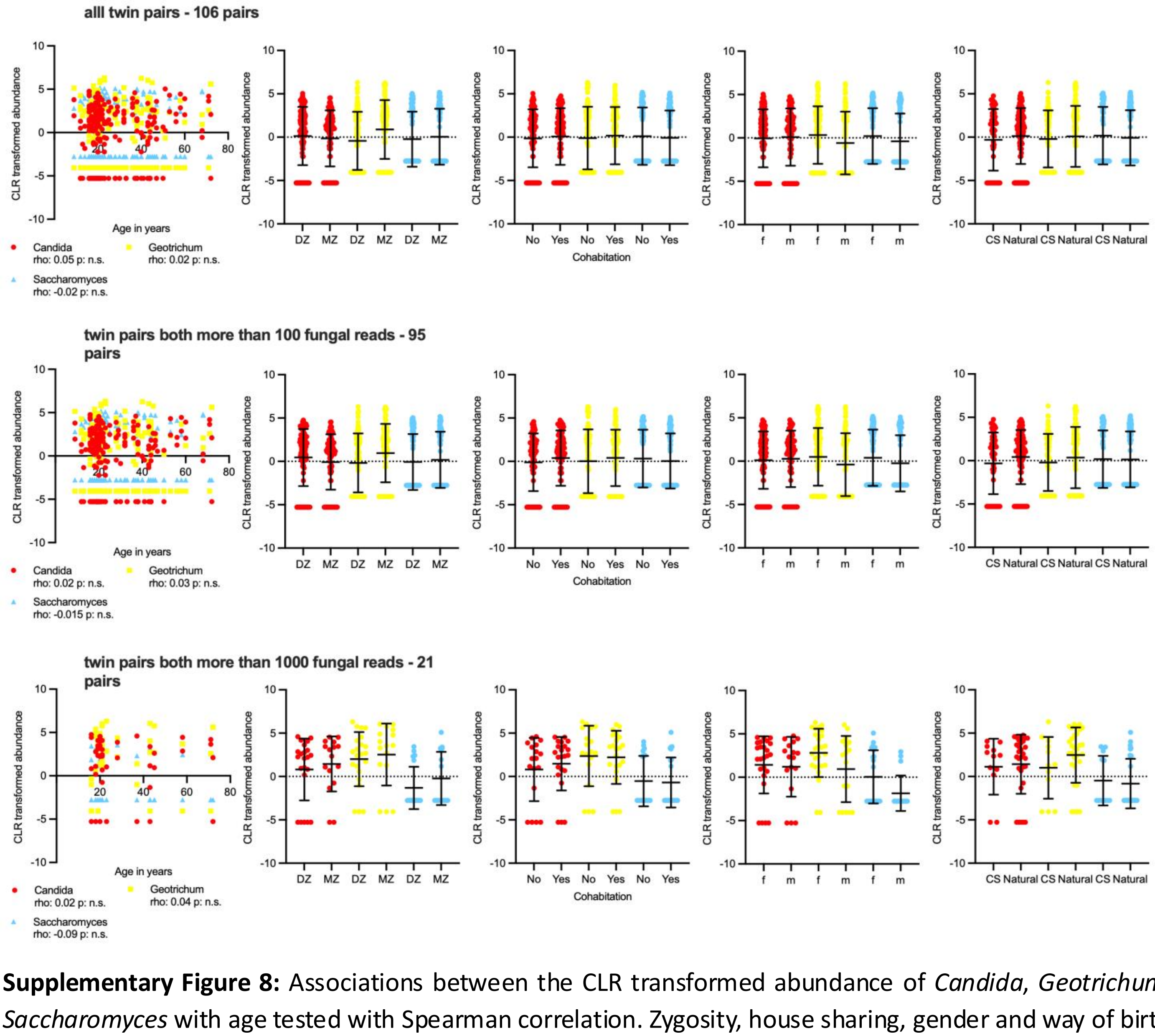

Supplementary Figure 8: Associations between the CLR transformed abundance of *Candida*, *Geotrichum*, *Saccharomyces* with age tested with Spearman correlation. Zygosity, house sharing, gender and way of birth tested with Mann-Whitney tests. Means +/- standard deviation are displayed. None statistical test reached significance level.

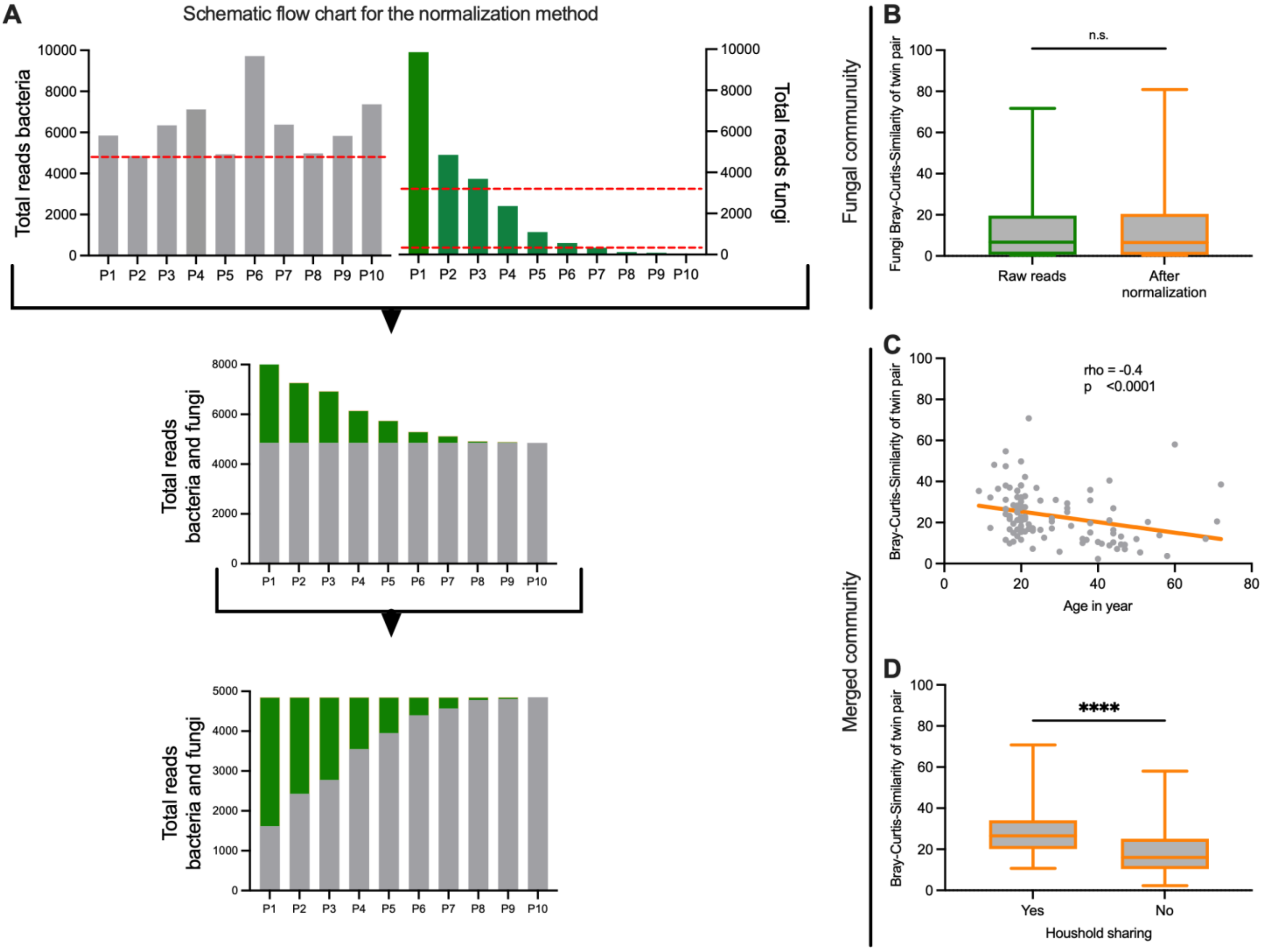

Supplementary Figure 9: (A) Schematic illustration of the bacterial and fungal community merging process. (B) Comparison of the Bray-Curtis similarity in twin pairs based on fungal reads before and after community merging. (C) Spearman correlation analysis between the Bray-Curtis resemblance of the merged community of a twin pair and the age of the twins. (D) Comparison of the Bray-Curtis similarities of the merged community of a twin pair with the houseshared status. Mann-Whitney-Test was used for testing statistical significance. The applied significance level nomenclature for all tests is: **** $p < 0.0001$. Box plot data are represented as mean +/- standard deviation (B and D).

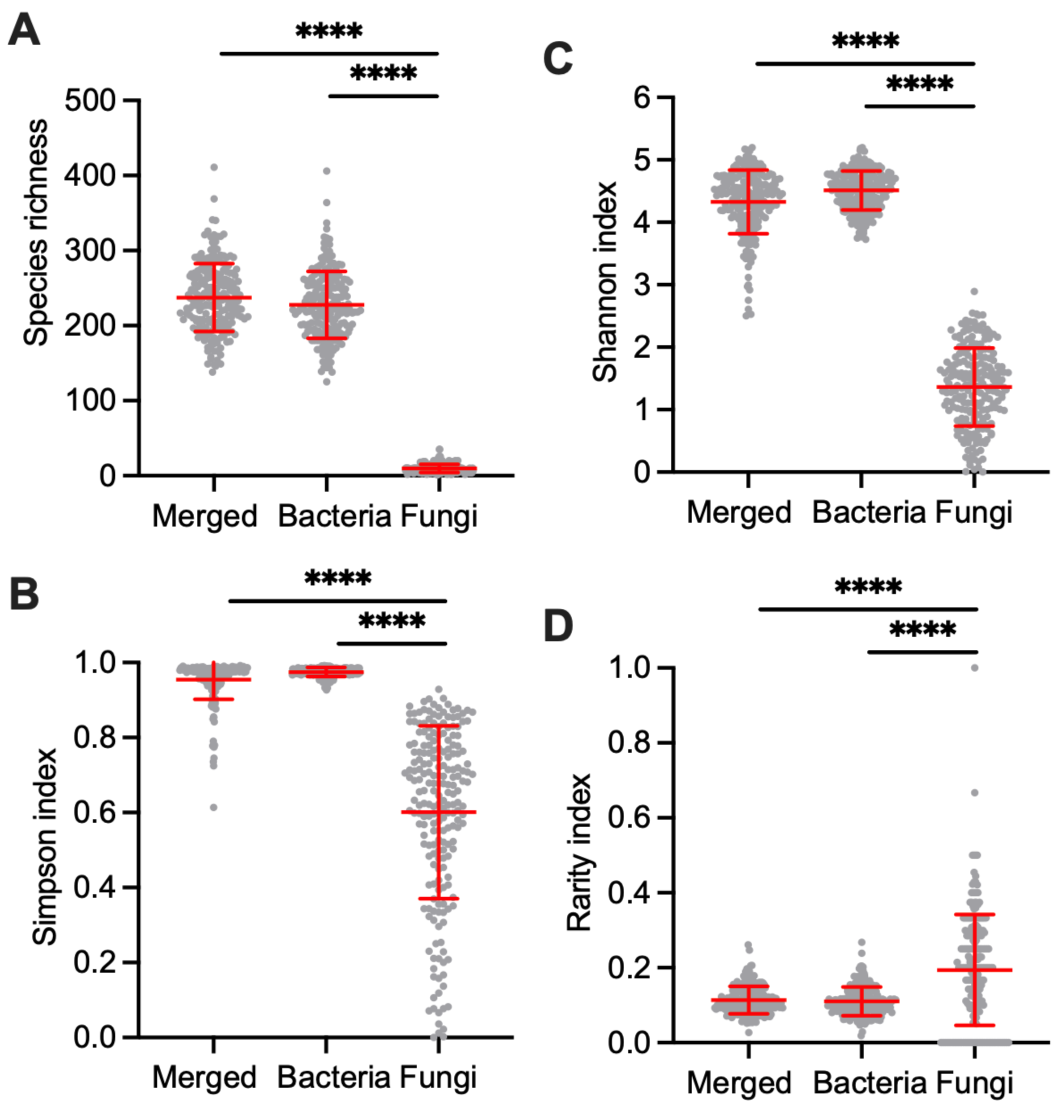

Supplementary Figure 10: The merged community of fungi and bacteria in mono- and dizygotic twins. Species richness (A), Simpson index (B), Shannon index (C), Rarity index (D) are compared for the bacterial, fungal, and merged community. Means +/- standard deviation are displayed. Mann-Whitney-Test was used for testing statistical significance. The applied significance level nomenclature for all tests is: **** $p < 0.0001$.

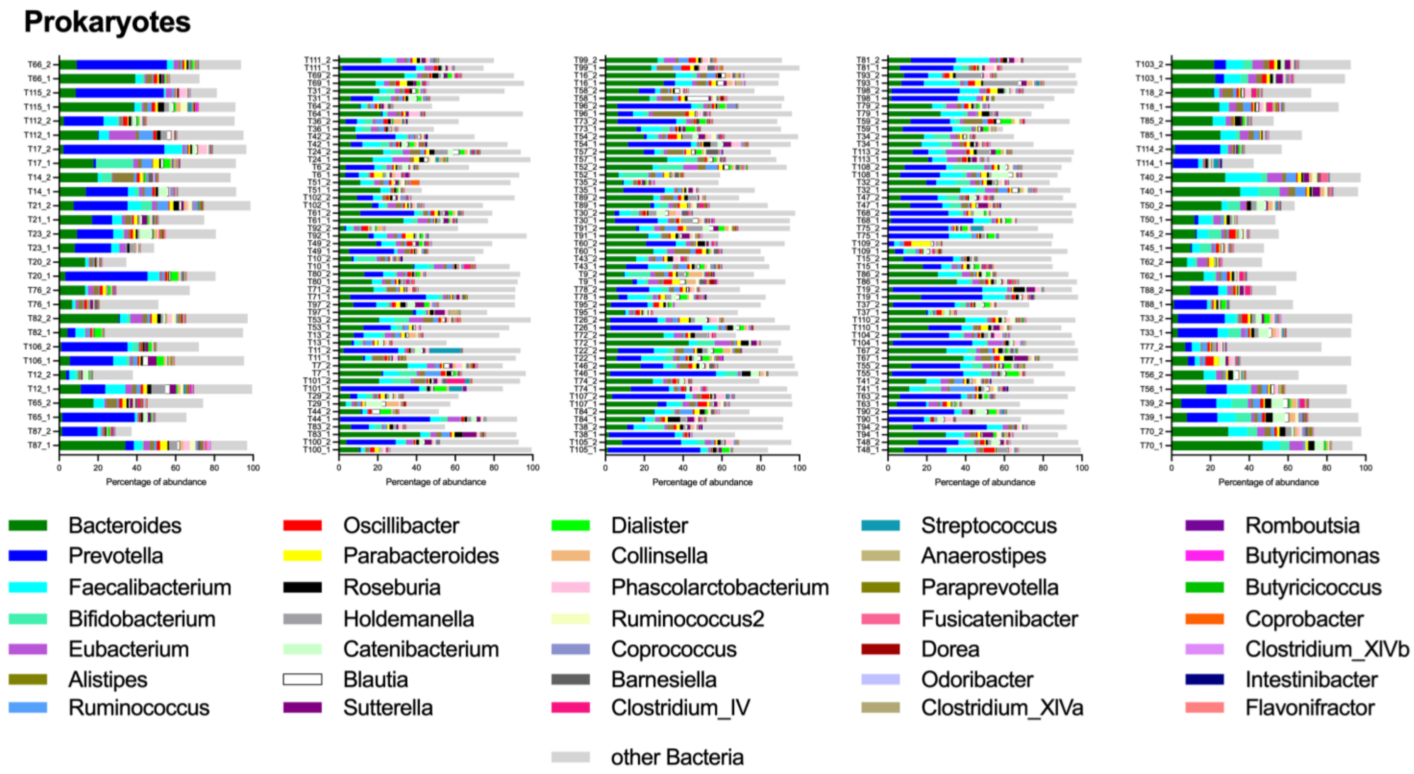

Supplementary Figure 11: Relative abundance of all bacterial genera, which are present in 50% of the cohort are shown for 212 individuals (106 twin pairs), respectively. The sum of other bacteria is added. Samples from twin pairs are sorted in increasing Bray-Curtis similarity (in percent).

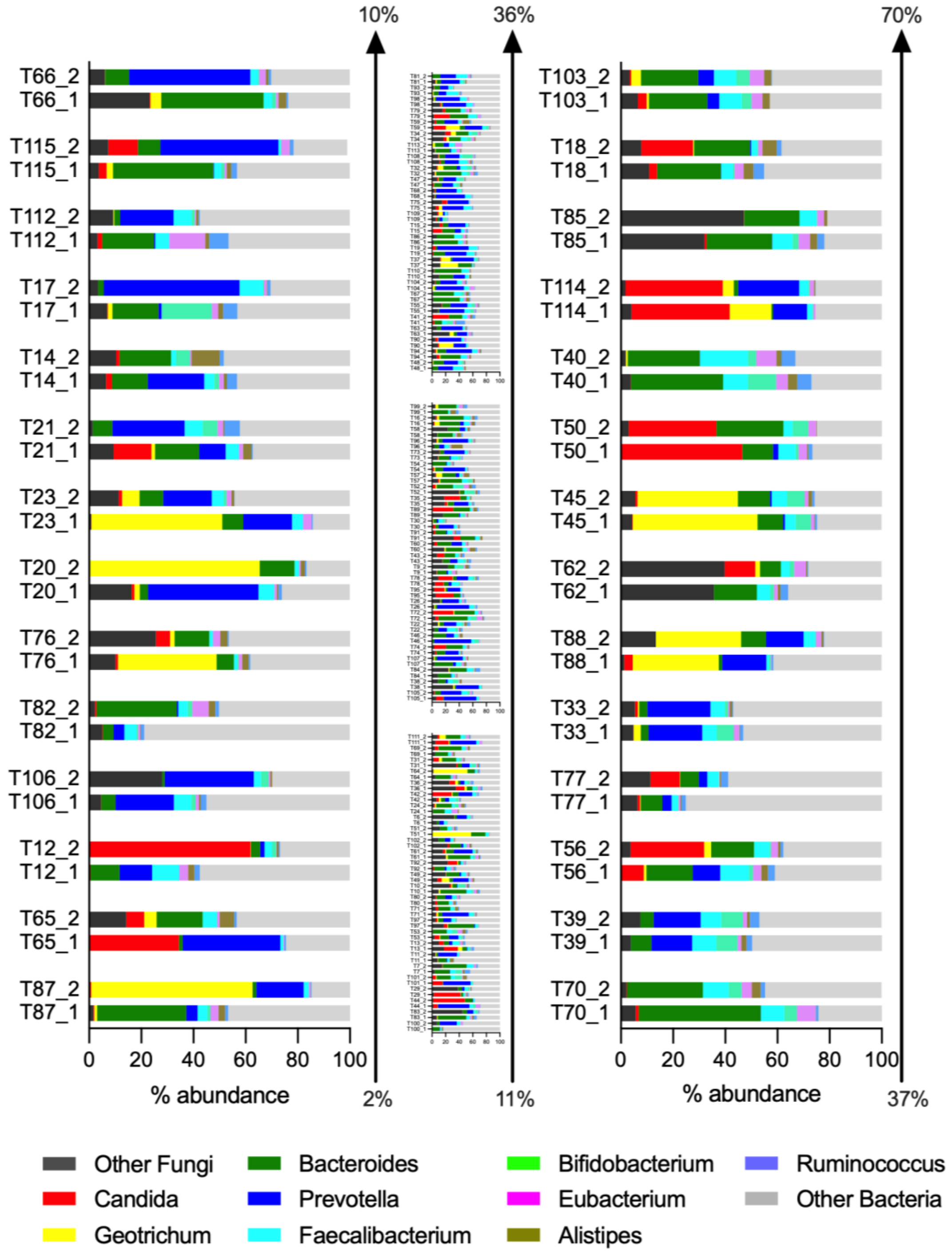

Supplementary Figure 12: Relative abundance of dominant bacteria and fungi in the cohort. Only those taxa present in more than 50% of the cohort (106 samples) and with more than 2% abundance on average are shown. Samples from twin pairs are sorted in increasing Bray-Curtis similarity.

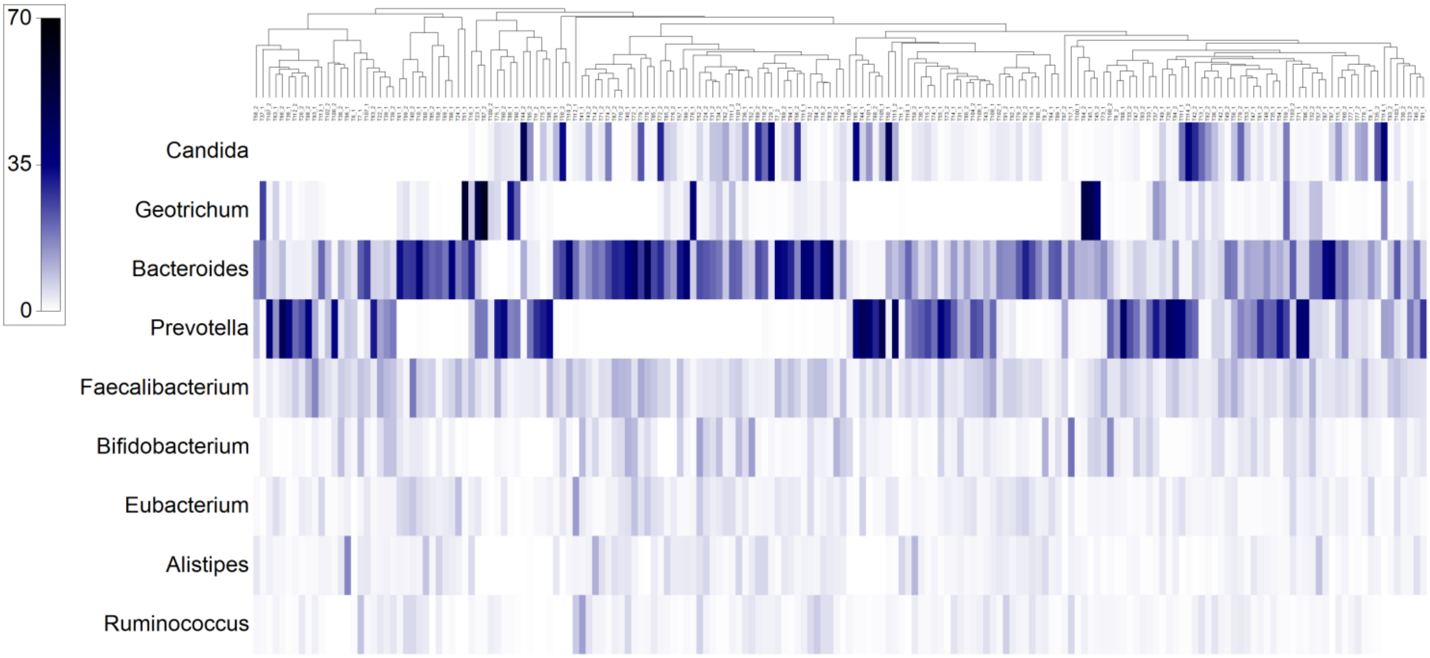

Supplementary Figure 13: Heatmap showing the percentage of abundance of the major microbial community clustered based on the CLR transformation and Euclidian similarity of the sample.

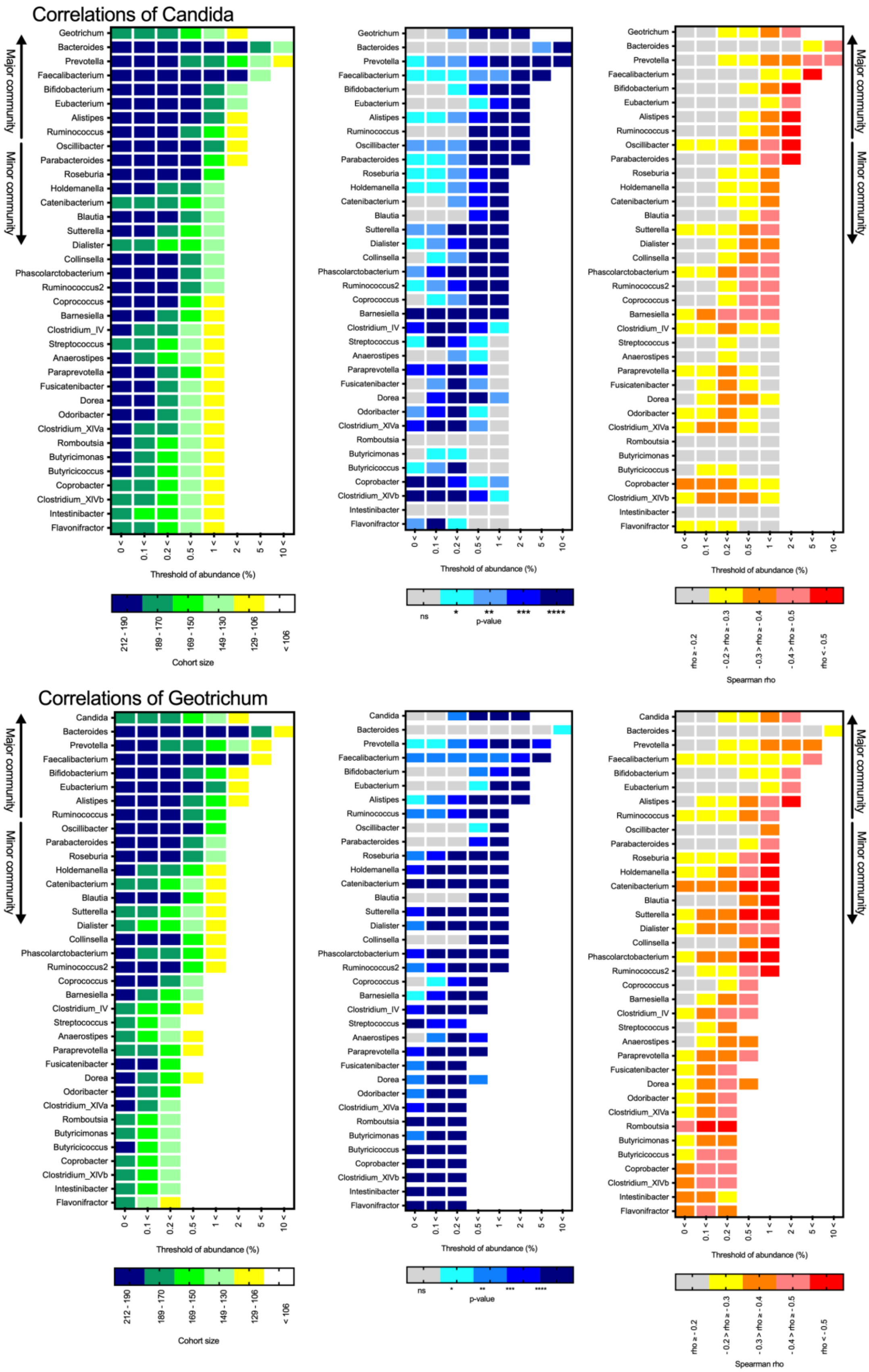

Supplementary Figure 14: Heatmap-like plots of Candida (up) and Geotrichum (down) across all bacterial genera present in 106 individuals (y-axis) and as a function of threshold % abundance (x-axis). The color visualizes the number of samples with co-occurrence of the genus pair, the Spearman correlation value rho of the genus pair, and the corrected p-value of this correlation. All values are shown only when the co-occurrence of the genera was present in 106 samples (50% of the cohort). Major microbial community has average abundance of bigger than 2%.

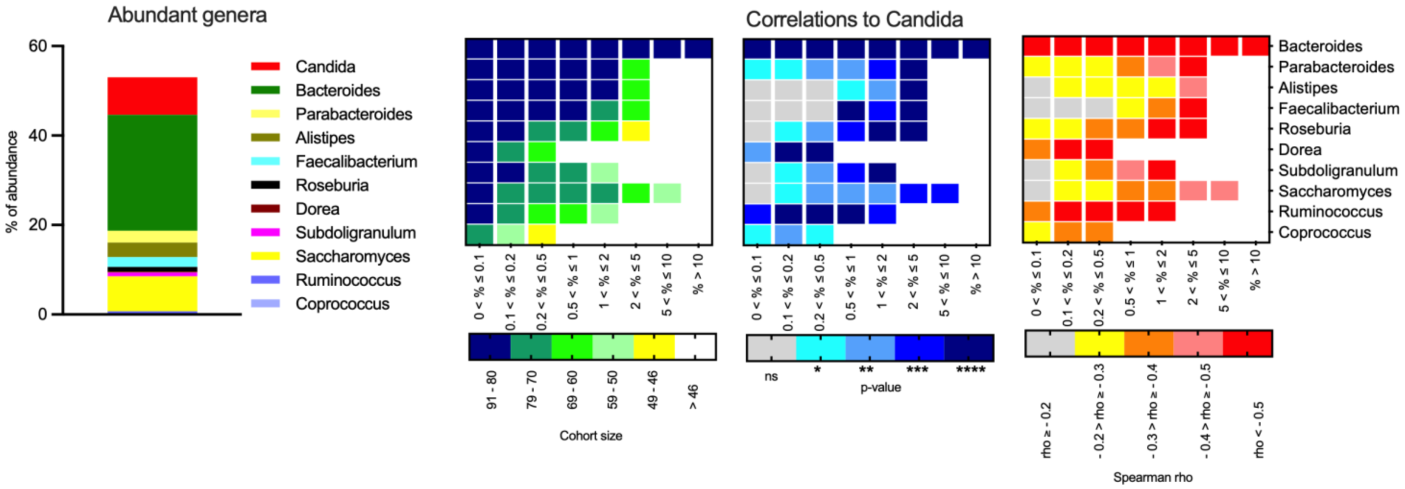

Supplementary Figure 15: Methodology validation with the publicly available abundance data from Hoffmann et al. Average relative abundance of dominant genera (present in more than 50% of the cohort). Heatmap-like plots of Candida across most dominant bacterial genera (y-axis) and as a function of threshold % abundance (x-axis). The color visualizes the number of samples with co-occurrence of the genus pair, the Spearman correlation value rho of the genus pair, and the corrected p-value of this correlation. The applied significance level nomenclature for all tests is: * p < 0.05, ** p < 0.01, *** p < 0.001, **** p < 0.0001. All values are shown only when the co-occurrence of the genera was present in 50% of the cohort.

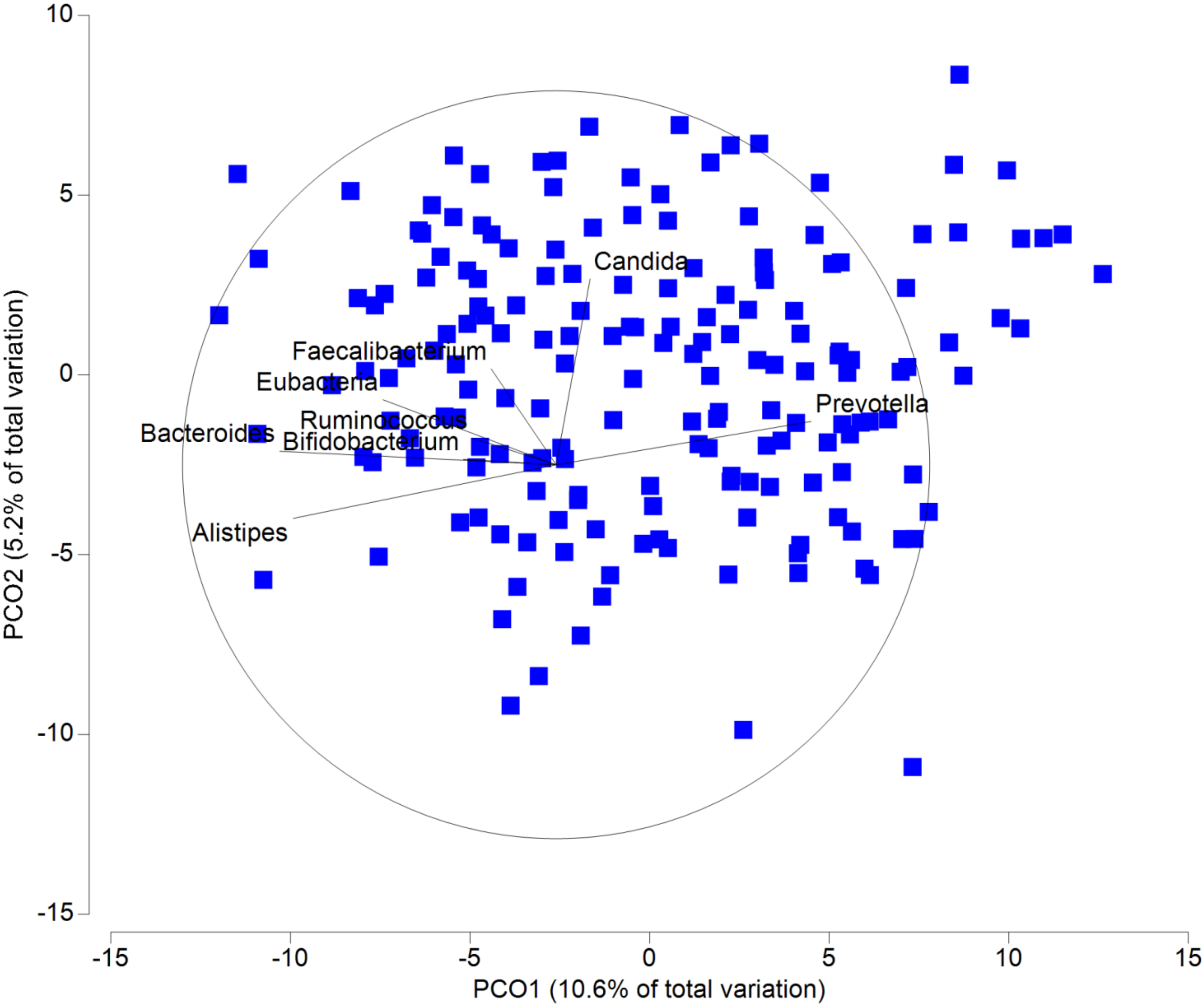

Supplementary Figure 16: PCO analysis of all genera present in 50% of the cohort based on CLR transformation and Euclidian similarity. Vectors of the major bacterial genera and *Candida* represent the distribution of genera.

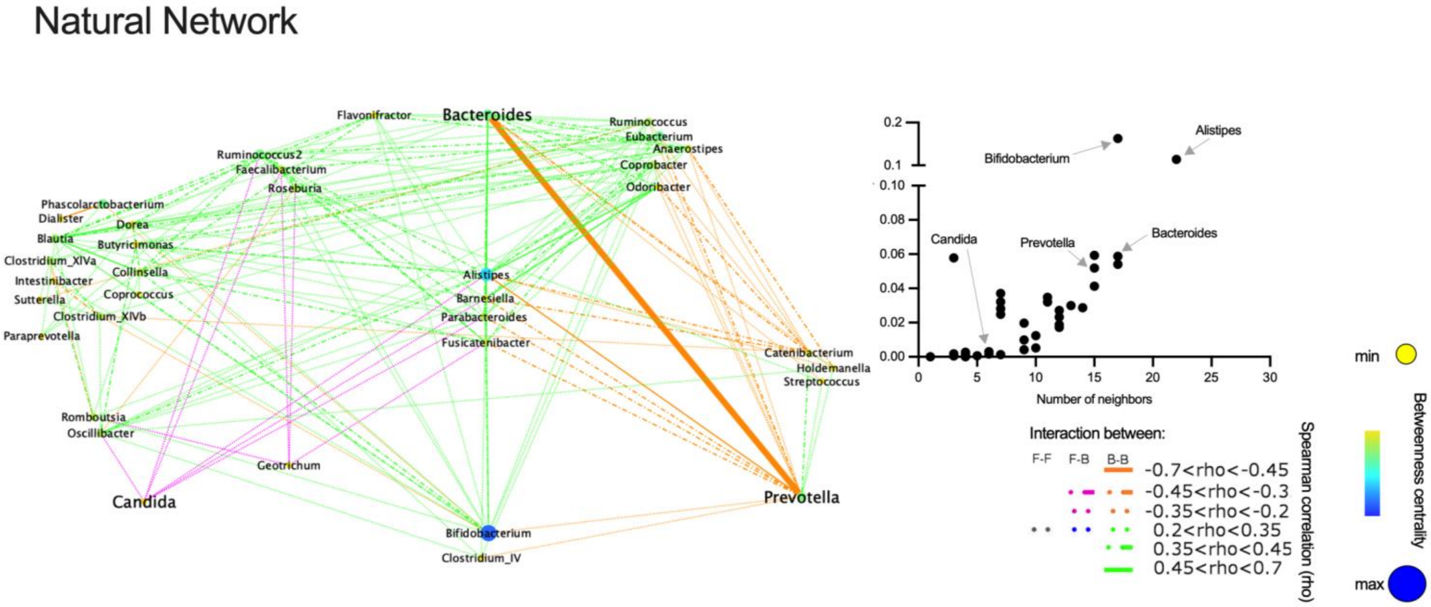

Supplementary Figure 17: Natural community network with the nodes representing the betweenness centrality. They are grouped by their interactions to the three most abundant taxa (Bacteroides, Prevotella, and Candida), all genera present in 106 individuals (50% of the cohort) were considered. Interactions show Spearman correlation (0.2 ≤ rho ≥ 0.2, corrected p-value<0.05). Betweenness centrality by number of neighbors is displayed next to it.

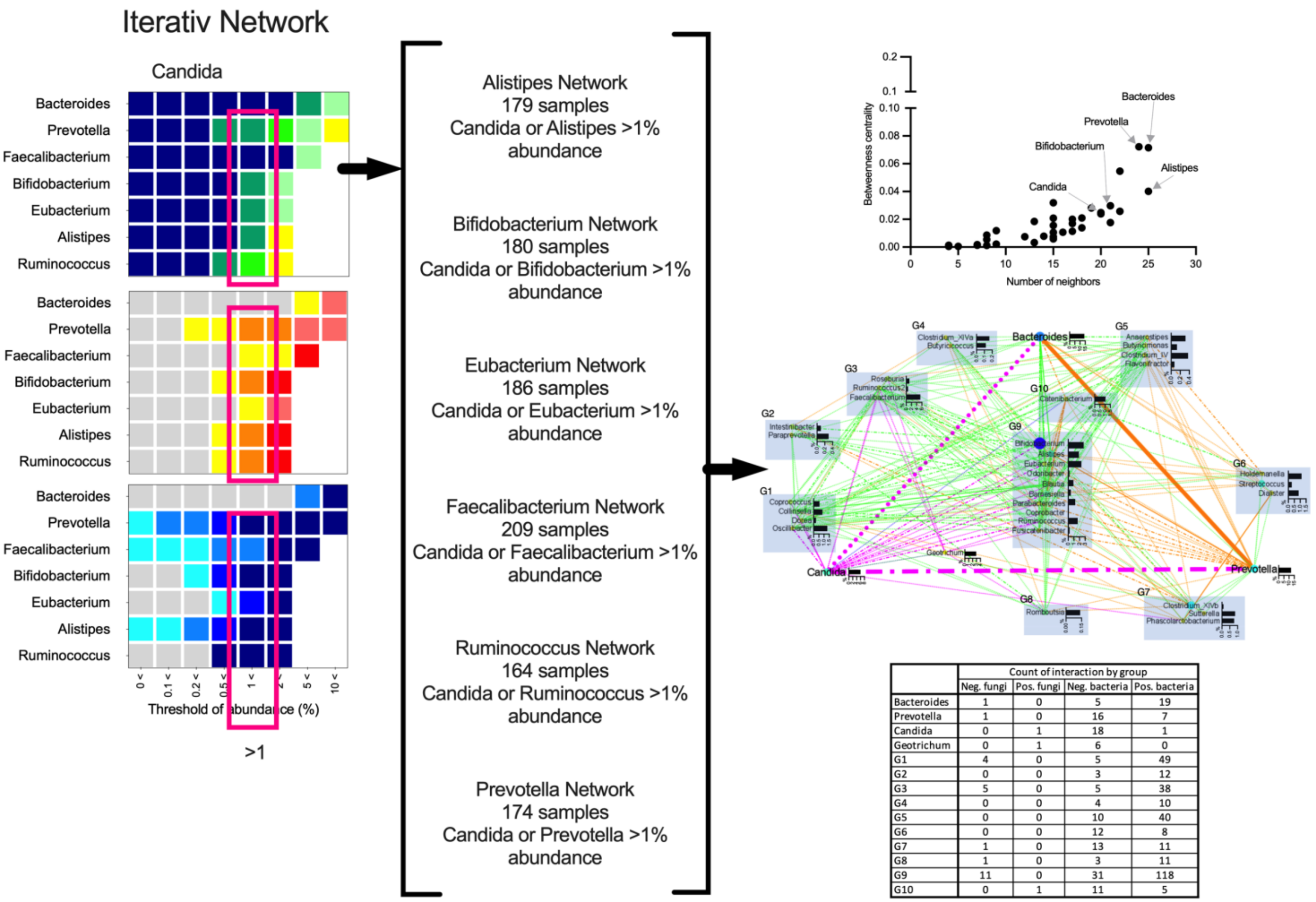

	Count of interaction by group			
	Neg. fungi	Pos. fungi	Neg. bacteria	Pos. bacteria
Bacteroides	1	0	5	19
Prevotella	1	0	16	7
Candida	0	1	18	1
Geotrichum	0	1	6	0
G1	4	0	5	49
G2	0	0	3	12
G3	5	0	5	38
G4	0	0	4	10
G5	0	0	10	40
G6	0	0	12	8
G7	1	0	13	11
G8	1	0	3	11
G9	11	0	31	118
G10	0	1	11	5

Supplementary Figure 18: Flow-chart for the generation of the iterative network together with interaction count and betweenness centrality by number of neighbors.

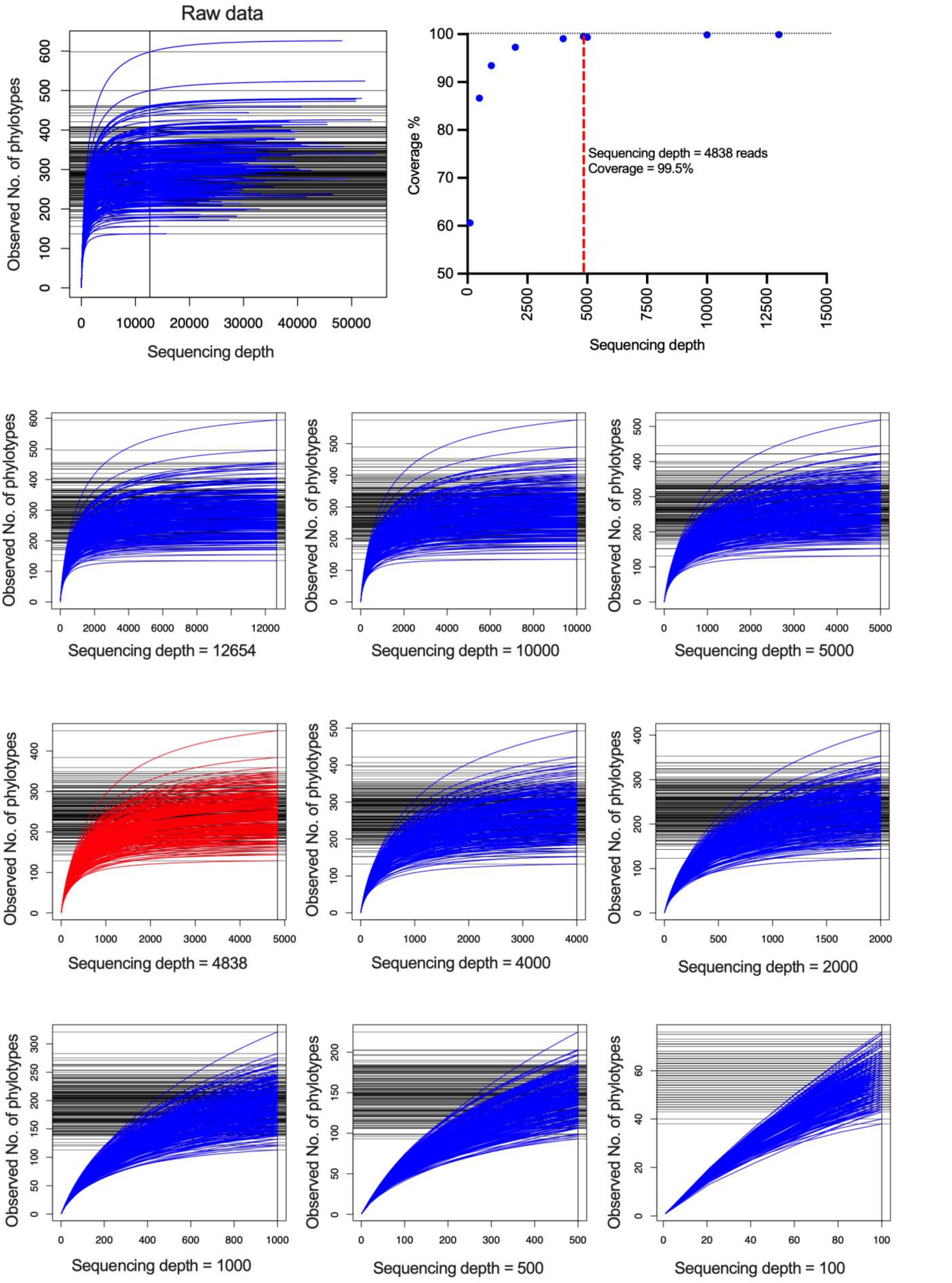

Supplementary File 19: Rarefaction curves and Good's coverage for different sequencing depths of the bacterial phylotype table.
